# Light-mediated multi-target protein degradation using arylazopyrazole photoswitchable PROTACs (AP-PROTACs)†

**DOI:** 10.1039/d2cc03092f

**Published:** 2022-09-01

**Authors:** Qisi Zhang, Cyrille S. Kounde, Milon Mondal, Jake L. Greenfield, Jennifer R. Baker, Sergei Kotelnikov, Mikhail Ignatov, Christopher P. Tinworth, Leran Zhang, Daniel Conole, Elena De Vita, Dima Kozakov, Adam McCluskey, John D. Harling, Matthew J. Fuchter, Edward W. Tate

**Affiliations:** Department of Chemistry, Imperial College London London W12 0BZ UK e.tate@imperial.ac.uk; Chemistry, School of Environmental & Life Sciences, the University of Newcastle, University Drive Callaghan NSW 2308 Australia; Department of Applied Mathematics and Statistics, Stony Brook University Stony Brook NY 11794 USA; Laufer Center for Physical and Quantitative Biology, Stony Brook University Stony Brook NY 11794 USA; GlaxoSmithKline, Medicines Research Centre Gunnels Wood Road Stevenage Hertfordshire SG1 2NY UK

## Abstract

Light-activable spatiotemporal control of PROTAC-induced protein degradation was achieved with novel arylazopyrazole photoswitchable PROTACs (AP-PROTACs). The use of a promiscuous kinase inhibitor in the design enables this unique photoswitchable PROTAC to selectively degrade four protein kinases together with on/off optical control using different wavelengths of light.

## Abstract 2

A new class of arylazopyrazole photoswitchable PROTACs (AP-PROTACs) enables light-triggered degradation of a specific ensemble of protein kinases.

## Graphical abstract

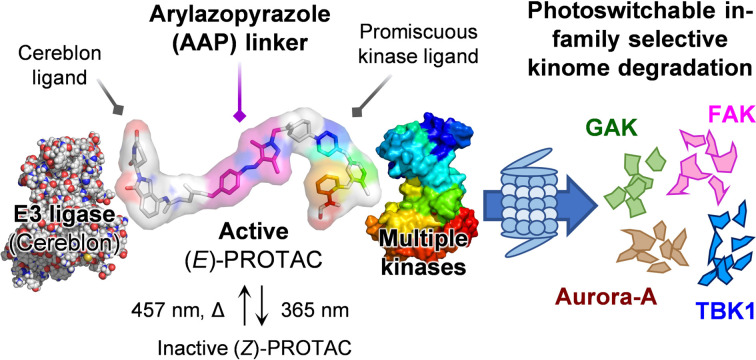

PROteolysis TArgeting Chimeras (PROTACs) are heterobifunctional small molecule degraders which recruit a protein of interest (POI) to a ubiquitin E3 ligase resulting in proteasomal degradation by the ubiquitin-proteasome system (UPS).^1,2^ By linking an E3 ligase ligand and a POI binder, PROTACs allow the formation of an E3 ligase/PROTAC/POI ternary complex which drives proximity-induced POI ubiquitination and subsequent degradation (Fig. 1A). Advantages including prolonged perturbation of protein expression, elimination of non-enzymatic functions, and access to novel tools for chemical protein knockdown have inspired widespread interest in this pharmacological paradigm.^3^ Potent small-molecule ligands for cereblon (CRBN) and Von Hippel–Lindau (VHL) E3 ligases, first used in PROTACs in 2015, have come to dominate the field,^4^ with CRBN-recruiting PROTACs entering clinical trials in 2019.

**Fig. 1:**
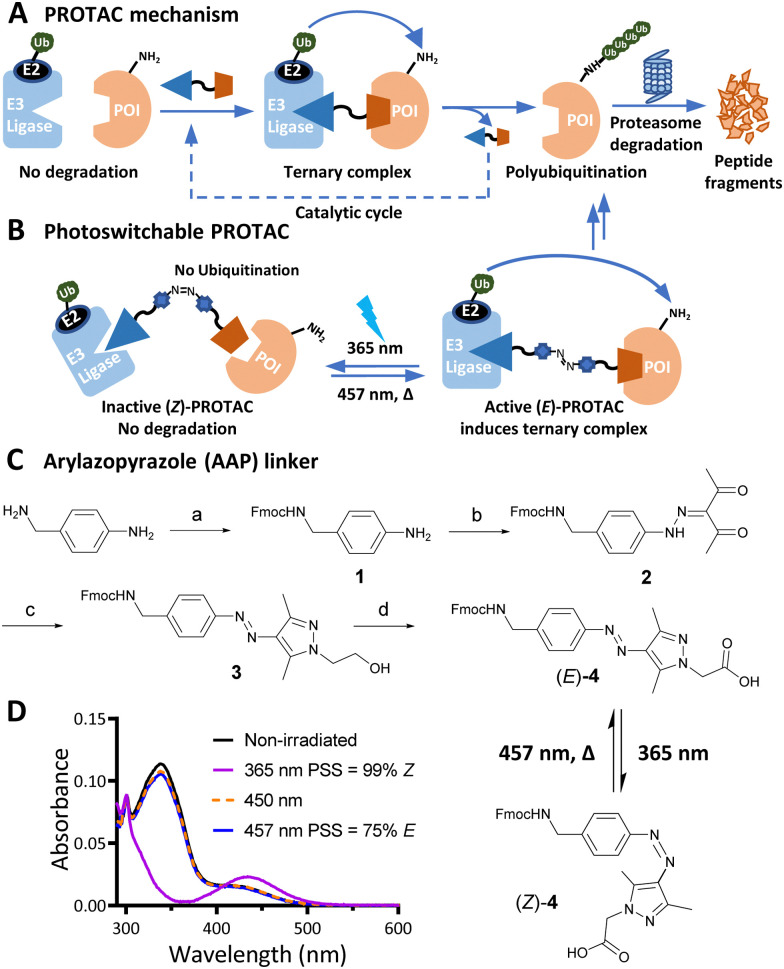
(A) PROTAC-mediated protein degradation mechanism. (B) Photoswitchable PROTACs allow control of targeted protein degradation; in the example shown the active *E* isomer induces ternary complex formation and protein degradation, until irradiated with 365 nm light to switch to the inactive *Z* isomer, which fails to form an effective ternary complex. (C) Synthetic route to arylazopyrazole (AAP) photoswitchable linker 4. Reagents and conditions: (a) Fmoc-OSu, DIPEA, DCM, RT, 16 h; 82%; (b) i. NaNO_2_, acetic acid, 37% HCl, 0 °C, 1 h; ii. acetylacetone, ethanol, sodium acetate, 0 °C to RT, 3 h; 83% over two steps; (c) 2-Hydrazinoethanol, DCM, MeOH, 50 °C, 2 h; 98%; (d) i. Dess–Martin periodinane, DMSO, RT, 16 h; ii. NaClO_2_, NaHPO_4_, 2-methylbut-2-ene, RT, 16 h; 40% over two steps. (D) Ultraviolet-Visible (UV-Vis) spectra of a 20 μM solution of 4 in water with 0.2% DMSO under the stated irradiation conditions.

Effective PROTACs have been developed against a wide array of protein targets, but spatiotemporal control of degradation remains much less explored. Conditional degradation may be achieved by selective targeting (*e.g.* through conjugation to an antibody) or by light-mediated photopharmacology.^5,6^ For example, caged PROTACs irreversibly initiate POI degradation through photocleavage of a blocking group,^7^ whilst photoswitchable PROTACs permit reversible tuning of protein degradation using light to switch between two distinct PROTAC isomers with different biological outcomes (Fig. 1B).^8^ A small number of photoswitchable PROTACs, also termed PHOtochemically TArgeting Chimeras (PHOTACs), have been reported to date, all of which incorporate an azobenzene photoswitch with either the *E* (*trans*) or *Z* (*cis*) isomer being more active than the alternate isomer in inducing protein degradation of a target protein, including FKBP12, BCR-ABL fusion protein, or BET proteins BRD2–4.^9–11^

Here, we report a new class of photoswitchable PROTAC incorporating a novel arylazopyrazole photoswitch (AP-PROTACs) featuring differentiated photoswitching properties, and the first example of non-azobenzene PHOTAC technology. With a bromodomain (BRD) ligand (AP-PROTAC-1) or a multi-kinase inhibitor (AP-PROTAC-2) as the PROTAC warhead, light-switchable degradation of multiple proteins was achieved. In particular, we demonstrate the first example of light-tuneable degradation of multiple specific protein kinases using a broad-spectrum kinase inhibitor as the POI ligand.

Arylazopyrazole (AAP) photoswitches are highly valued for their efficiency and high *Z* isomer thermal stability, but have yet to be exploited in a PHOTAC.^12,13^ A versatile *N*-Fmoc-protected AAP amino acid linker (4) was designed (Fig. 1C), which achieves a photostationary state (PSS) containing 75% *E* isomer following irradiation at 457 nm UV light, and can be switched to 99% *Z* isomer on irradiation at 365 nm, calculated by UV-visible absorption spectroscopy (Fig. 1D).^14^ Thalidomide or lenalidomide were used as generic CRBN recruiters, bearing short linkers at C4 of the phthalimide position, which is known to be compatible with CRBN binding.^15^

AP-PROTAC-1, incorporating pan-bromodomain inhibitor JQ1 as the POI ligand (Fig. 2A and Scheme S1, ESI†), achieved up to 78% *E* or 85% *Z* isomer at the PSS on irradiation with 457 nm or 365 nm light, respectively (Fig. 2B), and remained stable across 20 cycles of reversible switching with 365 nm and 457 nm irradiation (Fig. 2C). Minor differences observed in *Z* PSS for AP-PROTAC-1*versus* intermediate 4 were consistent with previous observations that substituents may influence photochemical behaviour (PSS ratio, switching wavelength, half-life).^12,16^ Considering these optical properties, in-cell switching of AP-PROTAC-1 was performed with intermittent irradiation for 1 min every 2 hours to maintain the PSS during the experiment. BRD4 and BRD2 protein levels were analysed by immunoblot after 6 h treatment of HeLa cells with 100 nM AP-PROTAC-1 irradiated at either 457 nm (*E*-enriched) or 365 nm (*Z*-enriched) (Fig. 2D). (*E*-enriched)-AP-PROTAC-1 showed higher degradation activity, whereby treatment achieved up to 86% BRD4 degradation compared to only 35% degradation with (*Z*-enriched)-AP-PROTAC-1. More strikingly, BRD2 levels were reduced by 75% with (*E*-enriched)-AP-PROTAC-1 but remained unaffected by treatment with the *Z*-enriched isomer. When tested at a higher concentration of 500 nM, AP-PROTAC-1 retained a differentiated degradation profile between 365 nm (*Z*-enriched) and 457 nm (*E*-enriched) although the degradation window between the two wavelengths was somewhat reduced (Fig. S1, ESI†). These encouraging initial results with AP-PROTAC-1 suggested that the azopyrazole photoswitch is a promising linker for light-responsive protein degraders.

**Fig. 2:**
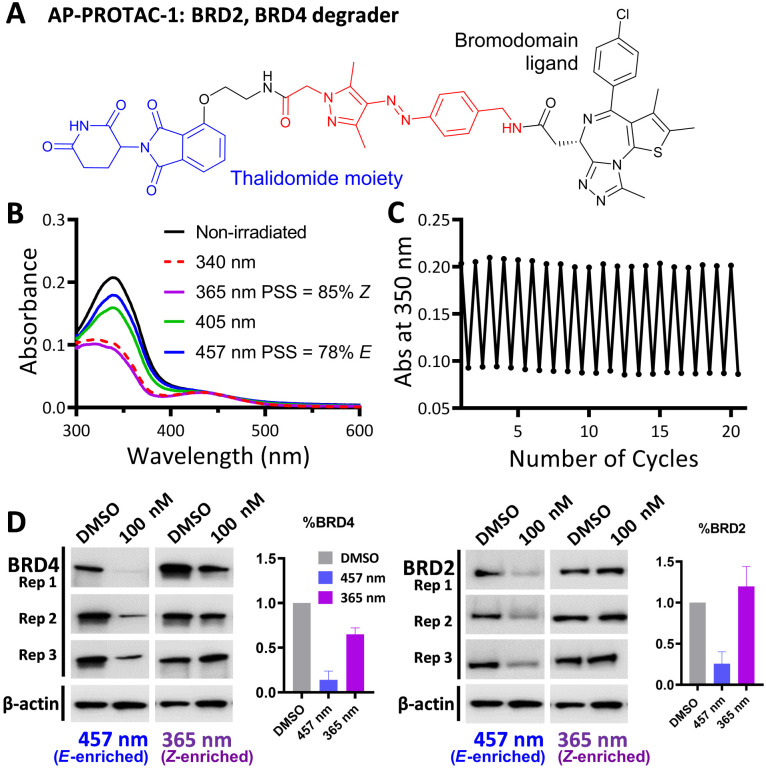
(A) Structure of AP-PROTAC-1. (B) UV-Vis spectra of a 20 μM solution of AP-PROTAC-1 in water with 0.2% DMSO with 3 min irradiation. (C) Reversible switching of a 25 μM solution of AP-PROTAC-1 in water with 0.25% DMSO following 365 nm or 457 nm irradiation for 20 cycles. (D) Immunoblot quantification of BRD4, BRD2 and β-actin in HeLa cells treated for 6 h with 100 nM pre-irradiated AP-PROTAC-1 with intermittent irradiation every 2 h at 457 nm or 365 nm. Bars represent signal normalized to β-actin, reported as the mean and SD of *n* = 3 biological replicates.

We next explored the extension of PHOTACs to switchable degradation of protein kinases, which are a key component of cellular signalling networks; over 85% of the human kinome (around 540 kinases in total) is dysregulated in at least one disease.^17^ To explore a wide range of potential targets in a single design, we designed a photoswitchable, multi-kinase targeting AP-PROTAC (AP-PROTAC-2) using a promiscuous kinase inhibitor. Previous (non-switchable) multikinase PROTAC designs have shown that a degrader may achieve higher and differentiated selectivity for target degradation over the parent POI ligand thanks to the more stringent requirement of active ternary complex formation *versus* target inhibition.^18,19^ To test our hypothesis that a photoswitch could add an additional layer of control, we selected kinase inhibitor CTx-0294885 as the POI ligand,^20^ due to its capacity to bind to 235 kinases across diverse kinase families.^21^AP-PROTAC-2 was synthesised from linker 4 over two sequential amide coupling reactions (Fig. 3A and Scheme S2, ESI†). UV-Vis spectroscopy (Fig. 3B) and LC–MS (Fig. 3C) revealed that AP-PROTAC-2 could be switched to 80% *E* isomer with 457 nm or to 90% *Z* isomer with 365 nm irradiation, with just 1 min irradiation sufficient to achieve PSS at 10 μM concentration (Fig. S2, ESI†). The thermal stability of the *Z* isomer at 37 °C was analysed by UV-vis spectroscopy and the half-life of the *Z* isomer was calculated as 31.7 h (Fig. 3D and Fig. S2, ESI†). Compared to previously reported PROTACs bearing diazo linkers, AP-PROTAC-2 shows comparable or improved PSS, faster switching, longer *Z* isomer half-life,^9–11^ and appeared stable across 20 switching cycles (Fig. 3E).

**Fig. 3:**
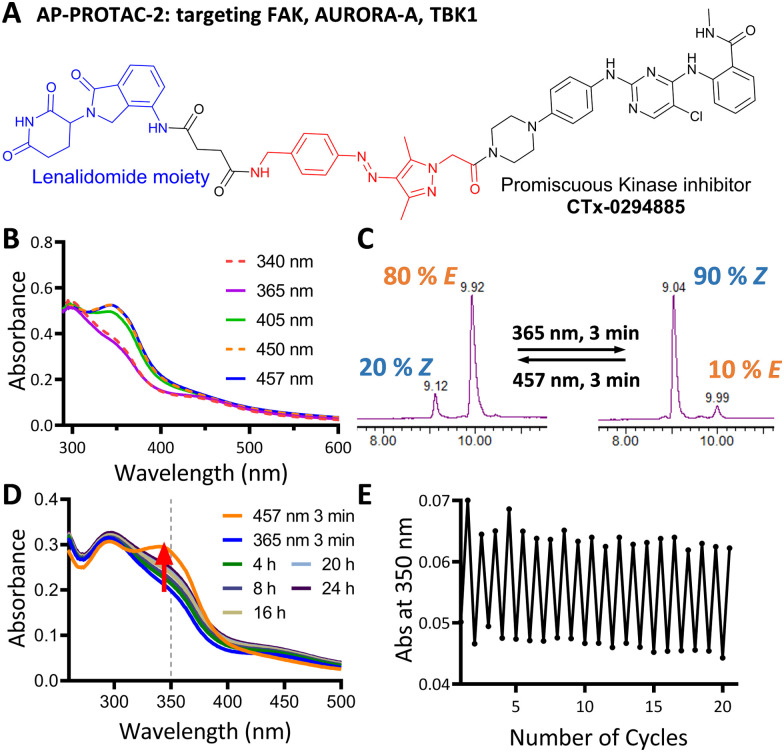
(A) Structure of Photoswitchable multi-kinase targeting degrader AP-PROTAC-2. (B) UV-Vis spectra of 20 μM AP-PROTAC-2 in water with 0.2% DMSO with 3 min irradiation. (C) LC-MS chromatograms of 0.1 mM AP-PROTAC-2 in 20% acetonitrile in water under the stated irradiation conditions. (D) UV-Vis spectra of 12 μM AP-PROTAC-2 in water with 0.1% DMSO after 365 nm irradiation recorded over time at 37 °C. (E) Reversible switching of 10 μM AP-PROTAC-2 solution in water with 0.1% DMSO at RT following 365 nm or 457 nm irradiation for 20 cycles.

Breast tumour cell line MDA-MB-231 was selected for cell experiments since *ca.* 75% of the kinome is known to be expressed in this line;^22^ cell viability assays at 100 nM or 500 nM AP-PROTAC-2 (pre-irradiated at 457 nm, *E*-enriched) showed no significant toxicity (Fig. S3, ESI†). Media with 100 nM AP-PROTAC-2 was irradiated for 3 min with 457 nm (*E*-enriched) or 365 nm (*Z*-enriched) light and used to treat cells for 24 h, in triplicate against 0.1% DMSO treatment control; to maximize *Z*/*E* isomer ratio, (*Z*-enriched)-AP-PROTAC-2 treated cells received intermittent irradiation for 1 min every 3 h for the first 17 h. Since which of the many diverse kinases bound by CTx-0294885 might be degraded by each isomer of AP-PROTAC-2 could not be determined *a priori*, we employed quantitative isobaric tandem mass tag (TMT) labelling proteomic analysis to measure the change in protein level for a total of 4,913 unique proteins including 159 kinases under each condition, in triplicate (Fig. 4).^23^ Kinase targets showing at least 30% reduction (log_2_(Fold Change) cut-off at −0.5) in protein level in treated samples compared to DMSO controls were prioritised for further analysis of isomer-dependent degradation (Fig. S4, ESI†). Remarkably, a total of just four kinases (2.5% of the detected kinome) were found to be significantly and consistently degraded by (*E*-enriched)-AP-PROTAC-2 but not by (*Z*-enriched)-AP-PROTAC-2, including GAK (Cyclin G-associated kinase), FAK (focal adhesion kinase), AURORA-A (Aurora kinase A) and TBK1 (TANK binding kinase 1) (Fig. 4, red dots).

**Fig. 4:**
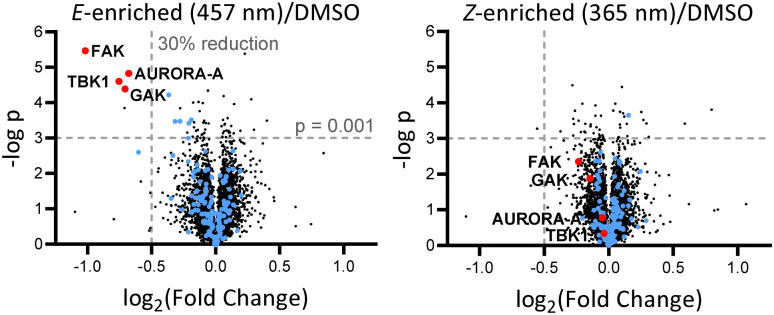
Volcano plots showing changes in protein level with AP-PROTAC-2 treatment. MDA-MB-231 cells were treated for 24 h with 100 nM AP-PROTAC-2 irradiated with 457 nm (*E*-enriched) or 365 nm (*Z*-enriched) (intermittent irradiation of *Z*-treated cells every 3 h for 1 min for the first 17 h) and the proteome analysed by multiplexed quantitative proteomics. The fold change in the relative abundance of 4,915 identified proteins comparing treated samples and the DMSO control is plotted against significance, –log(*p*) (*n* = 3, false discovery rate (FDR) <5%). Blue dots: protein kinases identified. Red dots: significantly and differentially affected kinases.

To find optimal conditions for isomer-selective degradation, we further explored these findings by immunoblot in MDA-MB-231 cells treated with 100 nM (*E*-enriched)-AP-PROTAC-2 or (*Z*-enriched)-AP-PROTAC-2 for 4, 8, 12 or 16 hours (Fig. 5A). In line with initial proteomics data, whilst (*E*-enriched)-AP-PROTAC-2 degraded 57% FAK and 69% AURORA-A by 16 h, (*Z*-enriched)-AP-PROTAC-2 was much less active, degrading FAK only up to 35% and AURORA-A up to 27% (Fig. 5C). However, no significant degradation was observed for GAK or TBK1 at 100 nM (Fig. S5, ESI†). In dose-response analyses undertaken at 16 h treatment (Fig. 5B), (*E*-enriched)-AP-PROTAC-2 achieved up to 85% D_max_ for both FAK and AURORA-A, and up to 70% for TBK1, at 300 nM, while (*Z*-enriched)-AP-PROTAC-2 required 500 nM to achieve up to 43% FAK and 47% AURORA-A degradation, and no TBK1 degradation (Fig. 5D and Fig. S6, ESI†). Through co-treatment with the proteasome inhibitor bortezomib we further verified that FAK, TBK1 and AURORA-A degradation by (*E*-enriched)-AP-PROTAC-2 is dependent on proteasome activity (Fig. S7, ESI†). Degradation of GAK was not observed under any tested condition using a commercially available GAK antibody, so degradation of this kinase could not be confirmed by immunoblot.

**Fig. 5:**
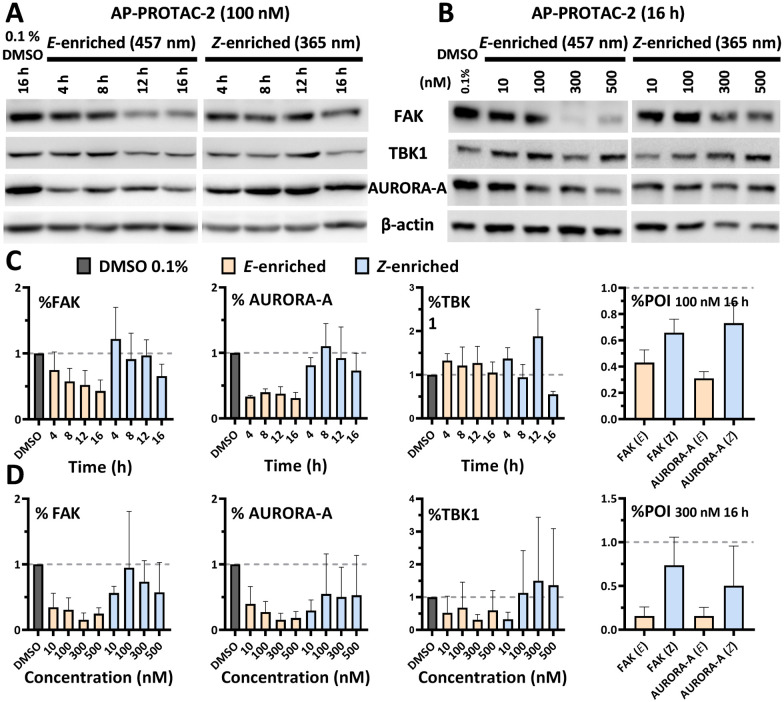
AP-PROTAC-2 differentially degrades FAK, AURORA-A and TBK1. (A) Immunoblots and (C) quantification of FAK, AURORA-A, and β-actin in MDA-MB-231 cells after 4 h, 8 h, 12 h, or 16 h treatment of 100 nM AP-PROTAC-2 irradiated with 457 nm (*E*-enriched) or 365 nm (*Z*-enriched) (with intermittent 365 nm irradiation every 3 h for *Z*-enriched flasks). (B) Immunoblots and (D) quantification of 16 h treatment of indicated concentrations of compounds. Bars represent mean signal normalized to β-actin, reported as the mean and SD of *n* = 3 biological replicates.

We further probed the putative ternary complex of (*E*) or (*Z*)-AP-PROTAC-2 with FAK and CRBN by fast Fourier transform (FFT)-based docking using PIPER with an additional “silent” convolution term used to find energetically favourable ternary complex poses.^24,25^ The overall population of feasible low-energy complex conformations formed with the *E* isomer was found to be significantly larger than for the *Z* isomer (Fig. 6), consistent with the enhanced ternary complex formation and empirical degradation efficiency driven by the *E* isomer.^26^

**Fig. 6:**
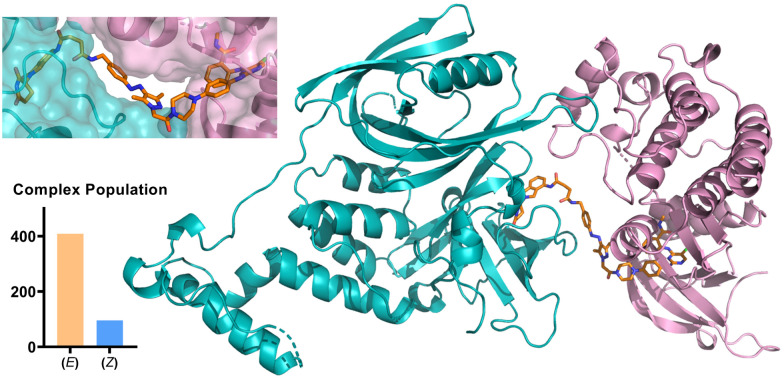
Structural model of CRBN/(*E*)-AP-PROTAC-2/FAK complex. Cyan – E3 ligase CRBN; pink – FAK; orange stick – (*E*)-AP-PROTAC-2. Bottom left: Overall population of feasible low-energy complex conformations formed with the *E* isomer *vs. Z* isomer of AP-PROTAC-2.

In summary, the first arylazopyrazole PROTACs (AP-PROTACs) demonstrate that AAP photoswitchable linker 4 has the potential to be applied in bifunctional compounds as a ‘plug and play’ switch to control a biological outcome. AP-PROTACs offer good PSS isomer abundance, fast switching, long *Z* isomer half-life, and stable reversible switching for multiple cycles. The biological responses of *E* and *Z* isomers of AP-PROTAC-1 remain well-differentiated, particularly for *E*-selective BRD2 degradation, potentially due to amplified (so-called “catalytic”) pharmacology enabled by the PROTAC mode of action. AP-PROTAC-2 is the first example of a multi-target PROTAC with photoswitchable degradation activity. We hypothesise that the remarkably selective switchable degradation driven by AP-PROTAC-2 is due to a combination of altered affinity for kinase binding coupled with the capacity to form a catalytically competent kinase/AP-PROTAC-2/CRBN ternary complex. Notably, FAK and AURORA-A kinases are both potential therapeutic targets in cancer. AURORA-A is a mitotic kinase that is essential for cell cycle progression,^27^ whilst FAK plays important roles in tumour progression and metastasis.^28^ Several FAK-targeting PROTACs have been reported and some showed anti-cancer therapeutic potential thanks to the elimination of the non-enzymatic functions of FAK.^29^ In addition to introducing a new class of photoswitch for light-responsive PROTACs, this work suggests opportunities for future development of multi-targeted degraders with readily available promiscuous inhibitors to achieve enhanced or synergistic target degradation.

## Conflicts of interest

J. D. H. is an employee and shareholder of Glaxo-SmithKline (GSK). E. W. T. is a director and shareholder of Myricx Pharma Ltd.

## Supplementary Material

CC-058-D2CC03092F-s001

CC-058-D2CC03092F-s002
