# Supplementary material for: Light-mediated multi-target protein degradation using arylazopyrazole photoswitchable PROTACs (AP-PROTACs)
Source: Chem Commun (Camb). 2022 Sep 1;58(78):10933–6. doi: 10.1039/d2cc03092f (PMC9521323; doi:10.1039/d2cc03092f)

Electronic Supplementary Material (ESI) for Chemical Communications.
This journal is © The Royal Society of Chemistry 2022

Supporting Information for

Light-mediated multi-target protein degradation using arylazopyrazole photoswitchable PROTACs (AP-PROTACs)

Qisi Zhang,[a] Cyrille S. Kounde,[a] Milon Mondal,[a] Jake L. Greenfield,[a] Jennifer R. Baker,[b] Sergei Kotelnikov,[c,d] Mikhail Ignatov,[c,d] Christopher P. Tinworth,[e] Leran Zhang,[a] Daniel Conole,[a] Elena De Vita,[a] Dima Kozakov,[c,d] Adam McCluskey,[b] John D. Harling,[e] Matthew J. Fuchter[a] and Edward W. Tate*[a]

a. Department of Chemistry, Imperial College London, London, W12 0BZ, UK. E-mail: e.tate@imperial.ac.uk

b. Chemistry, School of Environmental & Life Sciences, the University of Newcastle, University Drive, Callaghan, NSW 2308, Australia.

c. Department of Applied Mathematics and Statistics, Stony Brook University, Stony Brook, NY 11794, USA.

d. Laufer Center for Physical and Quantitative Biology, Stony Brook University, Stony Brook, NY 11794, USA.

e. GlaxoSmithKline, Medicines Research Centre, Gunnels Wood Road, Stevenage, Hertfordshire SG1 2NY, UK

Table of Contents

Supplementary Figures 2

Supplementary Information: Chemistry 7

General methods 7

Abbreviations 7

Synthesis 7

NMR spectra 11

Supplementary Information: Biology 29

Cell culture and treatment 29

Cell Lysis 29

Immunoblotting 29

Proteomics 29

Cell toxicity assay 30

Supplementary Information: Photoswitching properties characterisation 31

General Methods 31

UV-Visible spectroscopy determination of PSS 31

LC-MS spectra 33

UV-Visible spectroscopy determination of thermal half-life 37

Supplementary Information: Computational modelling of PROTAC ternary complex 37

References 38

Author contributions 38

Acknowledgement 38

Original blots 39

Supplementary Figures

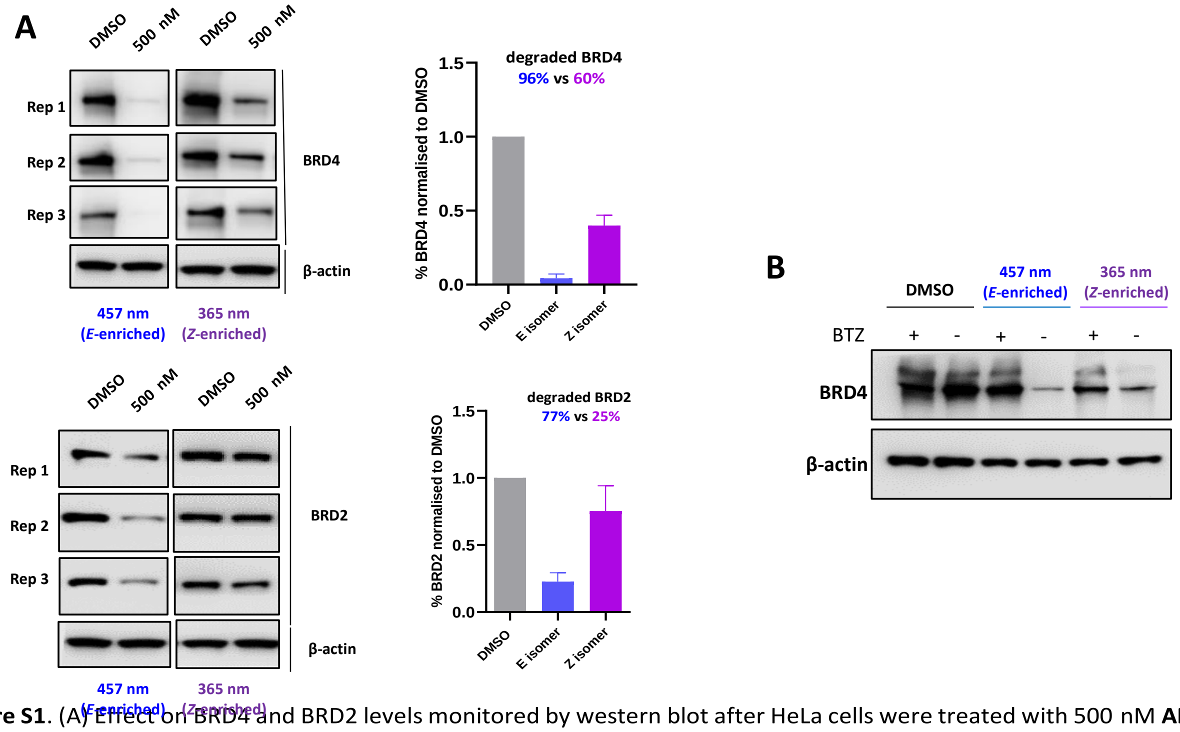

Figure S1. (A) Effect on BRD4 and BRD2 levels monitored by western blot after HeLa cells were treated with 500 nM **AP-PROTAC-1** and intermittently irradiated with 457 nm or 365 nm LED light every 2 h for 6 h. (B) Proteasome-dependency: HeLa cells were pre-incubated with proteasome inhibitor bortezomib (BTZ, 10 μM) for 2 h, then treated for 4 h with DMSO vehicle 0.1% (v/v) or 500 nM **AP-PROTAC-1** pre-irradiated at 457 nm or 365 nm.

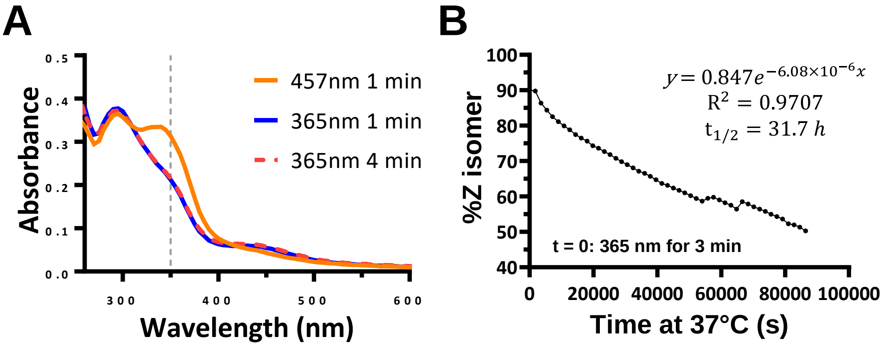

Figure S2. (A) UV–Vis spectra of 10 μM **AP-PROTAC-2** in water with 0.1% DMSO under the stated irradiation conditions. Irradiation of 365 nm light for 1 min is sufficient to achieve the photostationary state of 90% *Z* isomer in the solution. (B) A 12 μM **AP-PROTAC-2** solution in water was irradiated once with 365 nm light and kept at 37 ˚C. The change in absorbance at 350 nm was monitored by UV-Vis and the *Z* isomer percentage was plotted.

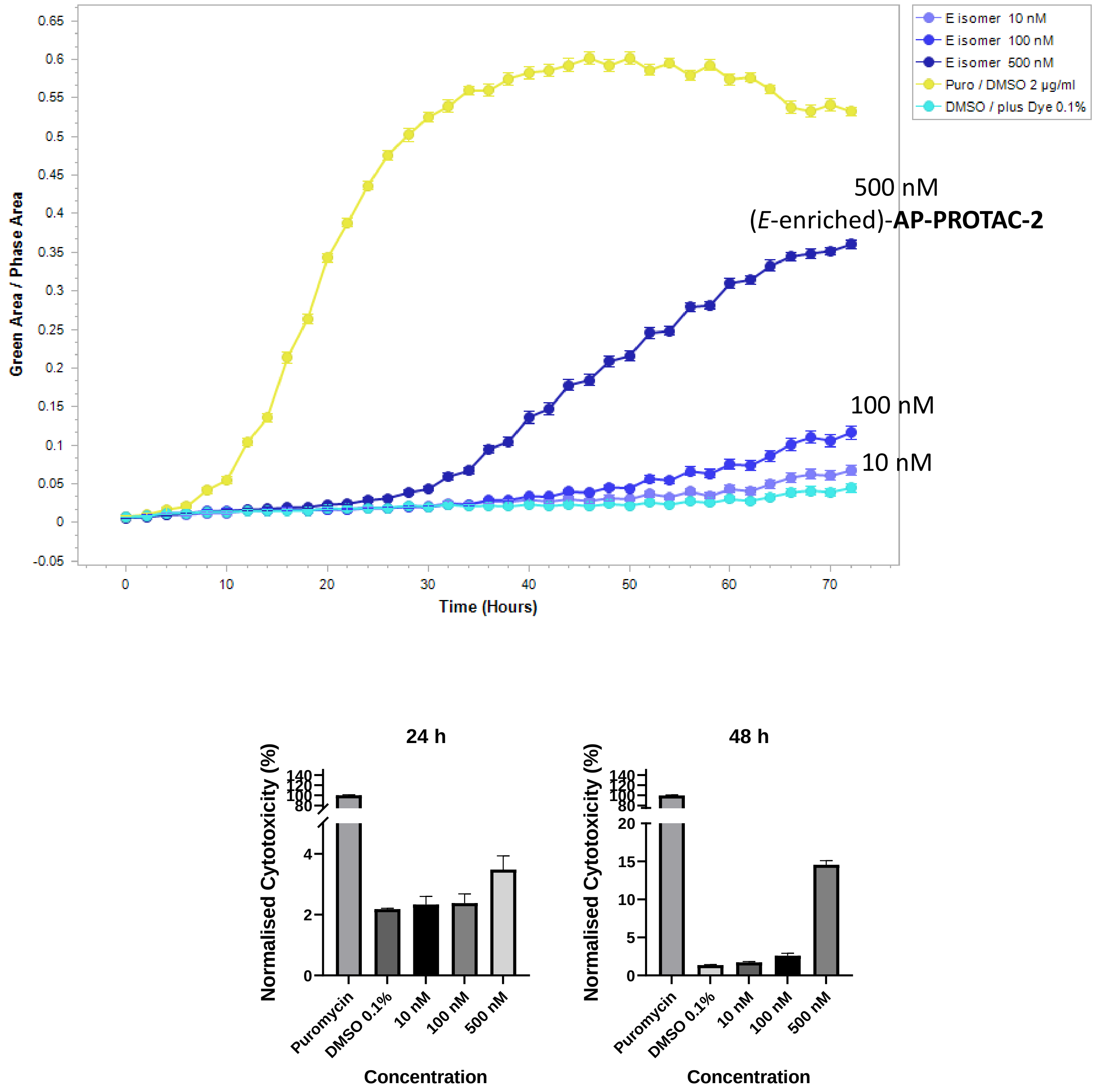

Figure S3. Cell viability assays of MDA-MD-231 cells treated with 10 nM, 100 nM, 500 nM (*E*-enriched)-**AP-PROTAC-2** or 0.1% DMSO and puromycin (2 μg/mL) as the controls. Top: plot of signal of SYTOX™ Green Nucleic Acid Stain over phase area for 72 h. Bottom: plots of signal at 24 h and 48 h normalised to puromycin death control.

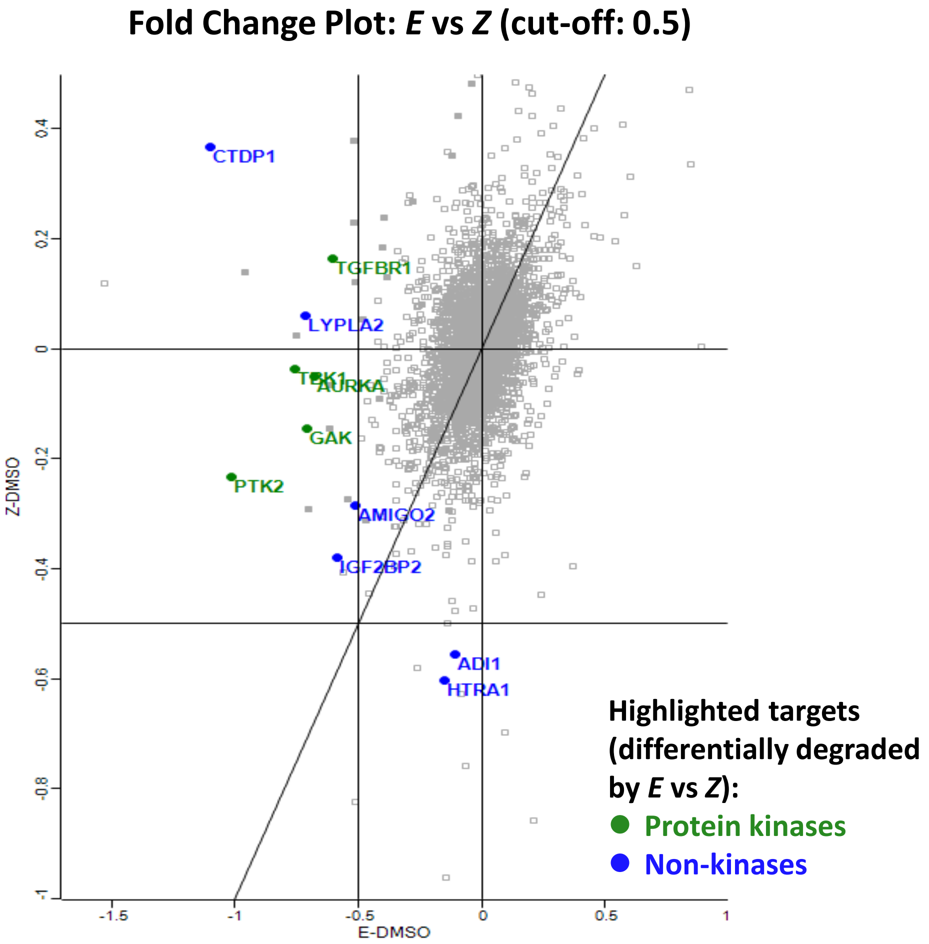

Figure S4. Fold change (FC) of (*E*-enriched)-**AP-PROTAC-2** treated sample versus DMSO control group (*E*-enriched − DMSO) plotted against Fold change of (*Z*-enriched)-**AP-PROTAC-2** treated sample versus DMSO control (*Z*-enriched − DMSO). Highlighted are possible proteins differentially degraded with (*E*-enriched) or (*Z*-enriched)-**AP-PROTAC-2** treatment.

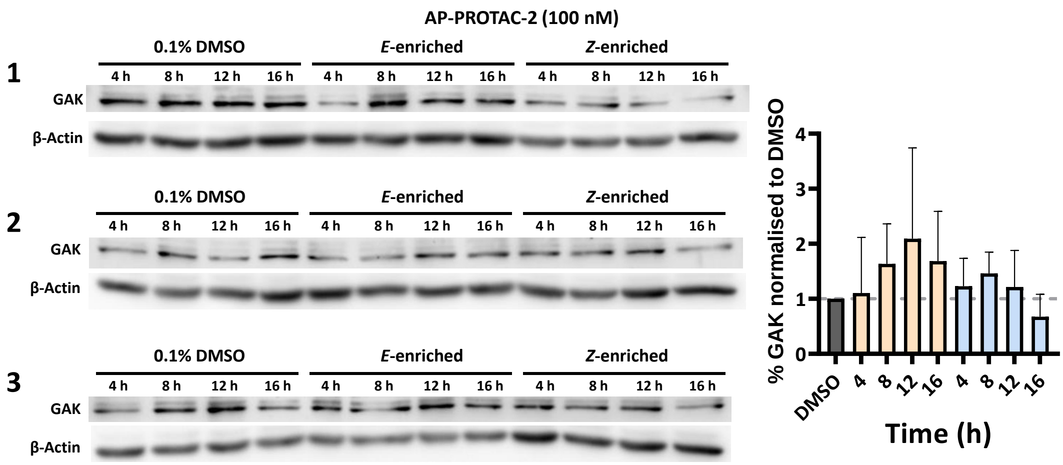

Figure S5. Immunoblots and quantification of GAK and β-actin in MDA-MB-231 cells after 4 h, 8 h, 12 h, or 16 h treatment of 100 nM (*E*-enriched)-**AP-PROTAC-2** or (*Z*-enriched) isomer (with intermittent irradiation every 3 h for *Z* isomer treated cells). Bars represent mean signal normalized to β-actin, reported as the mean and SD of n = 3 biological replicates.

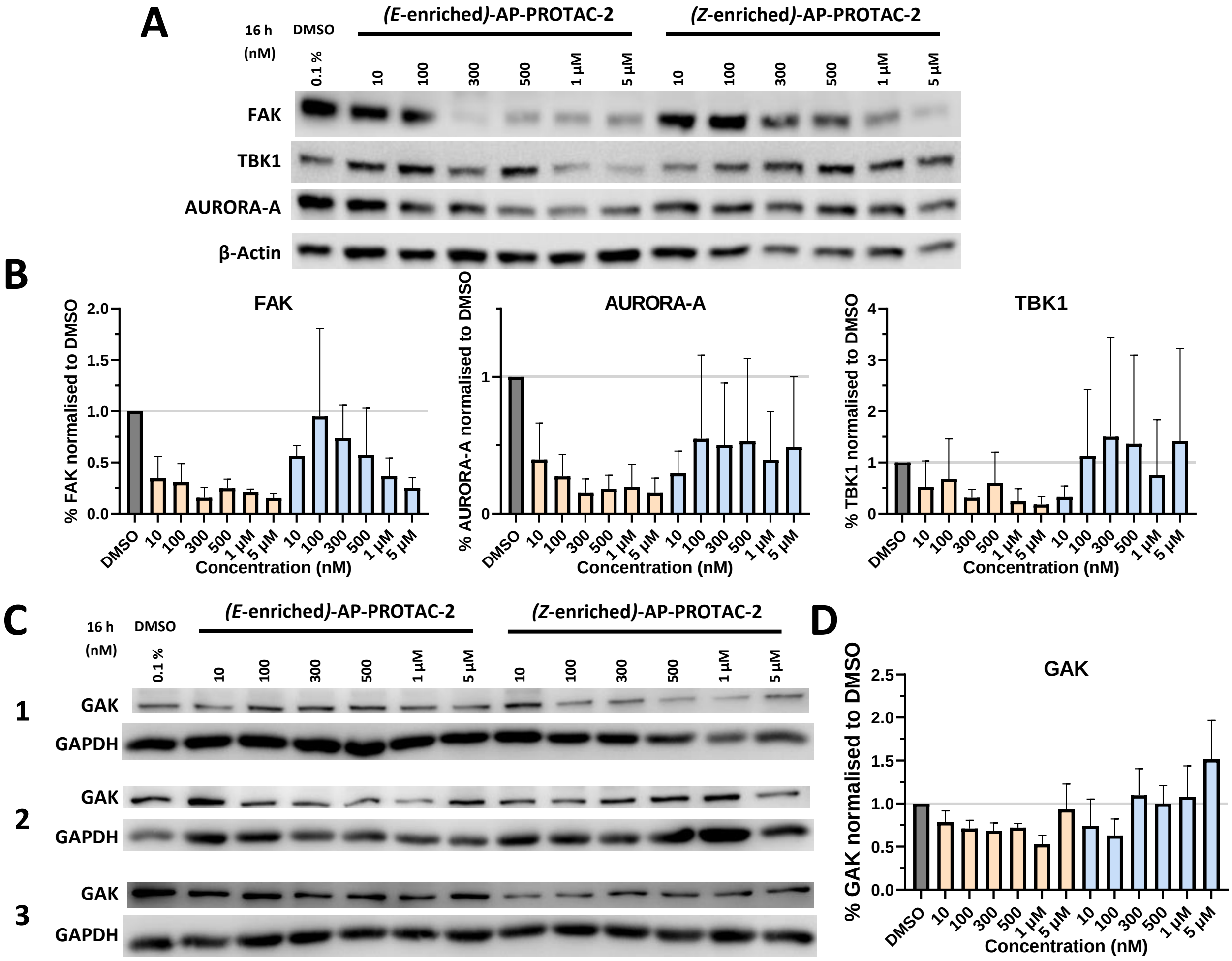

Figure S6. (A) Immunoblots and (B) quantification of FAK, TBK1, AURORA-A, and β-actin in MDA-MB-231 cells after 16 h treatment of indicated concentrations of compounds. (C) Immunoblots and (D) quantification of GAK, and β-actin in the same treatment. Bars represent mean signal normalized to β-actin, reported as the mean and SD of n = 3 biological replicates.

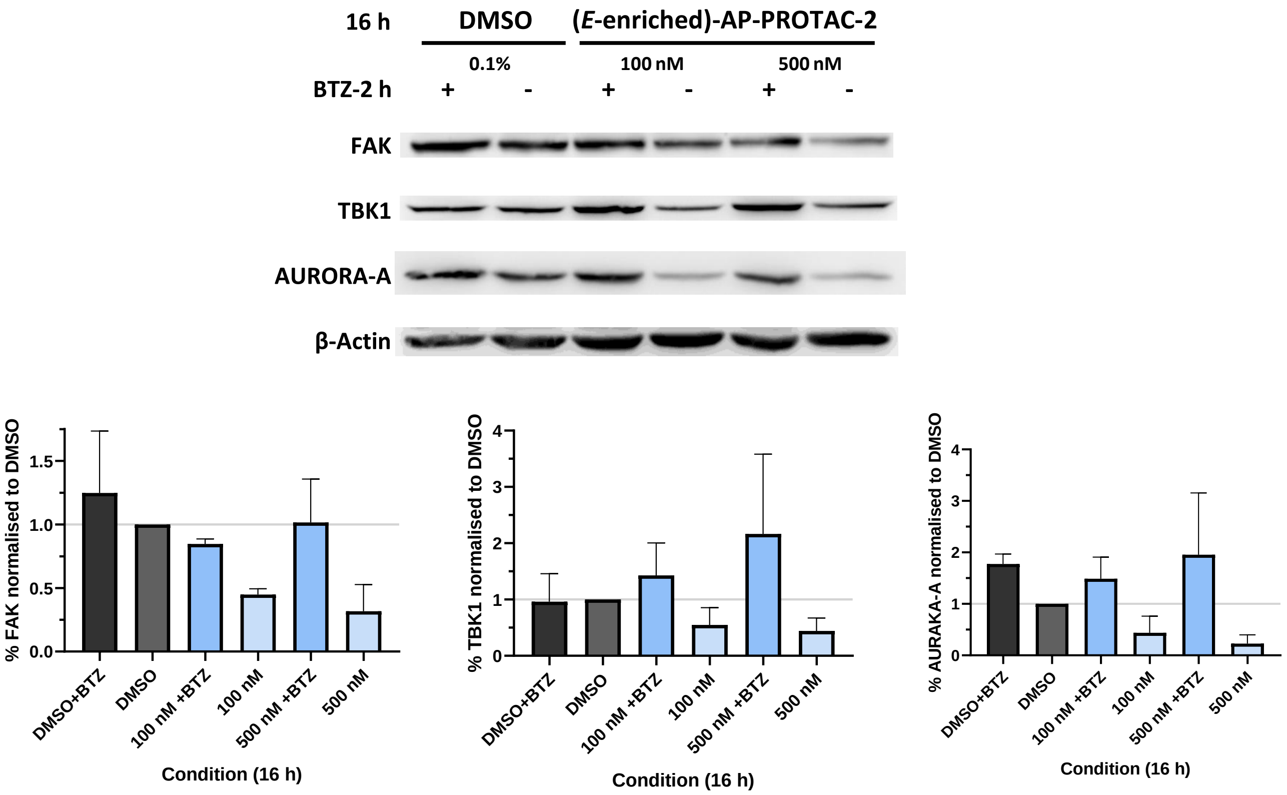

Figure S7. Immunoblots and quantification for FAK, AURORA-A TBK1, and β-actin in treated MDA-MB-231 cells. Cells were pre-treated for 2 h with or without 10 µM bortezomib (BTZ), and then treated with 0.1% DMSO, 100 nM or 500 nM of (*E*-enriched)-**AP-PROTAC-2** for 16 h. Bars represent mean signal normalized to β-actin, reported as the mean and SD of n = 3 biological replicates.

Supplementary Information: Chemistry

General methods

All reagents were purchased from commercial sources (Sigma-Aldrich, Merck, Fluorochem UK) and were used without further purification. Pan-BET bromodomain inhibitor JQ1-COOH were provided by GlaxoSmithKline Medicines Research Centre, Stevenage. Multikinase inhibitor CTx-0294885 were synthesised with flow chemistry approaches as previously described.[1]

Analytical thin-layer chromatography (TLC) on aluminium sheets with silica gel 60 F254 (Merck) were used and visualized with UV light (254 nm) or appropriate TLC stain to monitor reactions. Flash chromatography with silica gel Geduran Si 60 (0.040-0.063 mm, Merck) was performed for general compound purification. Nuclear Magnetic Resonance (NMR) spectra were recorded on a BRUKER AV-400 spectrometer at 400 MHz (^{1}H-NMR) and 101 MHz (^{13}C-NMR) in deuterated solvents at 298 K. Chemical shifts are given in parts per million (ppm), coupling constants J are given in Hertz, and spin multiplicities are given as s (singlet), d (doublet), t (triplet), q (quartet) or m (multiplet). Spectra were analysed with MestReNova.

Abbreviations

DCM: dichloromethane; DIPEA: diisopropylethylamine; DMF: dimethylformamide; DMP: Dess-Martin periodinane, 1,1,1-Tris(acetyloxy)-1,1-dihydro-1,2-benziodoxol-3-(1H)-one; DMSO: dimethyl sulfoxide; Fmoc: fluorenylmethyloxycarbonyl protecting group; HATU: 1-[Bis(dimethylamino)methylene]-1H-1,2,3-triazolo[4,5-b]pyridinium 3-oxide hexafluorophosphate; HRMS: high-resolution mass spectrometry; LC-MS: liquid chromatography-mass spectrometry.

Synthesis

(9H-fluoren-9-yl)methyl (4-aminobenzyl)carbamate (1)

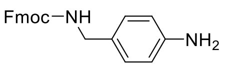

A solution of 4-(aminomethyl)aniline (1.39 ml, 12.3 mmol) in DCM (80.0 mL) was added DIPEA (2.14 mL, 12.3 mmol) and the mixture was cooled to 0 ˚C. A solution of 9-fluorenylmethyl *N*-succinimidyl carbonate (Fmoc-OSu, 4.14 g, 12.3 mmol) in DCM (30.0 mL) was slowly added. The reaction mixture was stirred for 16 h at room temperature. The resulting milky solution was washed with water (80.0 mL) and the organic layer was concentrated *in vacuo*. The crude was purified by flash column chromatography (30–80% ethyl acetate in n-hexane) to afford desired product **1** as a beige solid (3.50 g, 83%). **^{1}H NMR** (400 MHz, DMSO-*d$_6$*) δ 7.92 – 7.85 (m, 2H), 7.73 – 7.63 (m, 3H), 7.45 – 7.37 (m, 2H), 7.36 – 7.26 (m, 2H), 6.92 – 6.87 (m, 2H), 6.52 – 6.47 (m, 2H), 4.96 (s, 2H), 4.31 (d, *J* = 7.0 Hz, 2H), 4.21 (t, *J* = 6.9 Hz, 1H), 4.00 (d, *J* = 6.1 Hz, 2H). **^{13}C NMR** (101 MHz, DMSO-*d$_6$*) δ 156.2, 147.5, 143.9, 140.8, 128.1, 127.6, 127.0, 126.7, 125.2, 120.1, 113.7, 65.2, 46.8, 43.6. **HRMS** (ESI) m/z: calculated for $C_{22}H_{21}N_2O_2^+$, 345.1603; found 345.1590.

(9H-fluoren-9-yl)methyl(4-(2-(2,4-dioxopentan-3-ylidene)hydrazineyl)benzyl)carbamate (2)

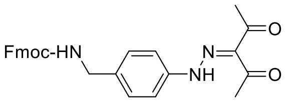

A solution of aniline **1** (500 mg, 1.45 mmol) in acetic acid (5.0 mL) was added HCl (1.40 mL, 37%) and the solution was cooled to 0 ˚C. A solution of sodium nitrite (200 mg, 2.90 mmol) in water (2.00 mL) was then added. The reaction mixture was stirred at 0 ˚C for 1 h, then a solution of acetylacetone (0.193 mL, 1.88 mmol) and sodium acetate (476 mg, 5.81 mmol) in ethanol (5.0 mL) was added. The bright yellow solution obtained was let warm to room temperature. After 3 h, the mixture was added ethyl acetate (20.0 mL) and washed with sodium bicarbonate (0.5 M, 3 × 10 mL) and brine (3 × 10 mL). The organic layer was dried over Na_2SO_4, filtered, and concentrated. The crude product was further purified by flash column chromatography (40 % ethyl acetate in hexane) to afford diketone **2** as an orange solid (442 mg, 67%). **^{1}H NMR** (400 MHz, $CDCl_3$) δ 14.75 (s, 1H), 7.83 – 7.76 (m, 2H), 7.65 – 7.59 (m, 2H), 7.47 – 7.26 (m, 8H), 5.23 (s, 1H), 4.51 (d, *J* = 6.8 Hz, 2H), 4.39 (d, *J* = 6.1 Hz, 2H), 4.24 (t, *J* = 6.7 Hz, 1H), 2.63 (s, 3H), 2.51 (s, 3H). **^{13}C NMR** (101 MHz, $CDCl_3$) δ 198.1, 197.2, 156.6, 143.9, 141.4, 141.0, 136.4, 133.3, 128.9, 127.8, 127.1, 125.1, 120.1, 116.6, 66.8, 47.4, 44.6, 31.8, 26.8. **HRMS** (ESI) m/z: calculated for $C_{27}H_{26}N_3O_4^+$, 456.1923; found 456.1919.

(9H-fluoren-9-yl)methyl(E)-(4-((1-(2-hydroxyethyl)-3,5-dimethyl-1H-pyrazol-4-yl)diazenyl)benzyl)carbamate (3)

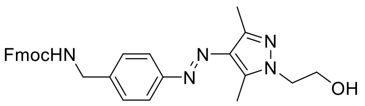

Diketone **2** (150 mg, 0.329 mmol) was dissolved in a mixture of DCM (5.0 mL) and methanol (5.0 mL). 2-Hydrazinoethanol (0.034 mL, 0.493 mmol) was added, and the mixture was stirred at 50˚C for 2 h. DCM (5.0 mL) and aq. HCl (1 M, 5.0 mL) were added to the crude mixture. The

extracted organic layer was washed with aq. HCl (1 M, 3 × 5.0 mL), dried over Na_2SO_4 and concentrated to yield alcohol **3** as a yellow solid (151 mg, 93%). **^{1}H NMR** (400 MHz, $CDCl_3$) δ 7.80 – 7.72 (m, 5H), 7.64 – 7.57 (m, 2H), 7.45 – 7.29 (m, 5H), 5.13 (s, 1H), 4.49 (d, *J* = 6.8 Hz, 2H), 4.44 (d, *J* = 6.1 Hz, 2H), 4.28 – 4.23 (m, 3H), 4.09 – 4.04 (m, 2H), 2.63 (s, 3H), 2.55 (s, 3H). **^{13}C NMR** (101 MHz, $CDCl_3$) δ 152.9, 144.0, 141.5, 141.3, 128.2, 127.9, 127.2, 125.1, 122.4, 120.2, 106.4, 61.4, 50.3, 47.5, 44.9, 19.2, 13.9, 10.0. **HRMS** (ESI) m/z: calculated for $C_{29}H_{30}N_5O_3^+$, 496.2349; found 496.2341.

(E)-2-(4-((4-(((((9H-fluoren-9-yl)methoxy)carbonyl)amino) methyl)phenyl)diazenyl)-3,5-dimethyl-1H-pyrazol-1-yl) acetic acid (linker 4)

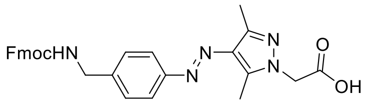

A solution of alcohol **3** (150 mg, 0.303 mmol) in DMSO (4.0 mL) was added Dess–Martin periodinane (DMP, 381 mg, 0.898 mmol). The solution was stirred for 16 h at room temperature. A solution of sodium chlorite (50 mg, 0.559 mmol) and NaH_2PO_4 (55 mg, 0.93 mmol) in water (0.5 mL) was added to the mixture, followed by. 2-methylbut-2-ene (0.1 mL, 0.93 mmol). The solution was stirred for 16 h at room temperature. Water (20.0 mL) was added, and the mixture was filtered to remove the DMP by-product. The filtrate was extracted with ethyl acetate (5 × 10 mL), and the combined organic layer was dried over Na_2SO_4, filtered, and concentrated *in vacuo*. The product was purified by flash column chromatography (3% – 7% methanol in DCM) and freeze-dried to yield linker **4** as a yellow solid (82 mg, 54%). **^{1}H NMR** (400 MHz, DMSO-*d$_6$*) δ 7.96 – 7.87 (m, 3H), 7.75 – 7.66 (m, 4H), 7.46 – 7.39 (m, 2H), 7.37 – 7.31 (m, 4H), 4.74 (s, 2H), 4.41 – 4.35 (m, 2H), 4.30 – 4.21 (m, 3H), 2.51 (s, 3H), 2.38 (s, 3H). **^{13}C NMR** (101 MHz, DMSO-*d$_6$*) δ 169.6, 156.9, 152.6, 144.4, 141.6, 141.2, 140.7, 135.0, 128.2, 128.1, 127.5, 127.5, 125.6, 121.8, 120.6, 65.8, 52.4, 47.3, 44.0, 14.3, 10.0. **HRMS** (ESI) m/z: calculated for $C_{29}H_{28}N_5O_4^+$, 510.2141; found 510.2128.

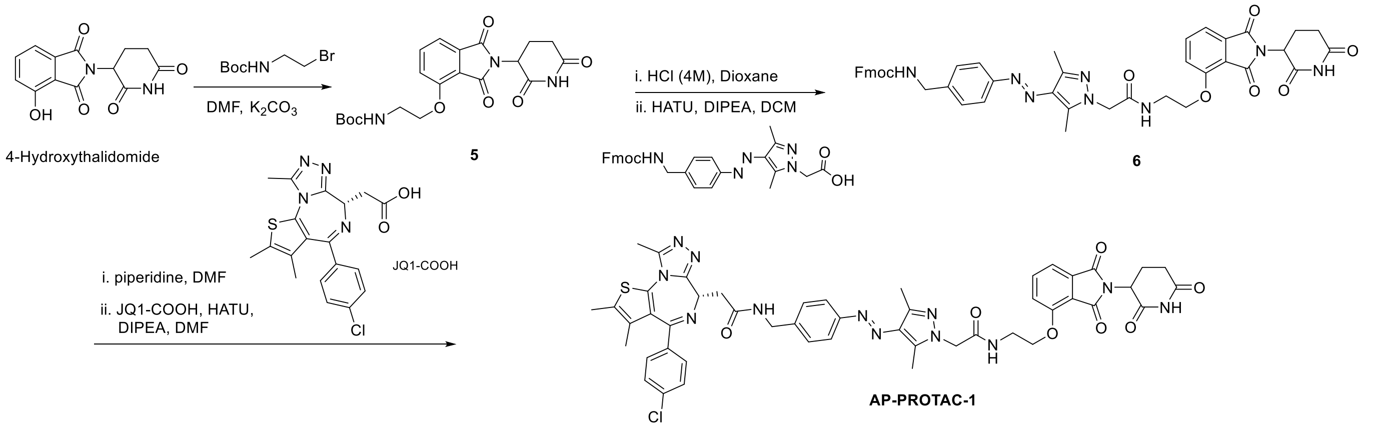

Scheme S1. Synthesis of BRD4-targeting photoswitchable **AP-PROTAC-1**.

Tert-butyl (2-((2-(2,6-dioxopiperidin-3-yl)-1,3-dioxoisoindolin-4-yl)oxy)ethyl)carbamate (5)

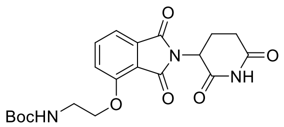

To a solution of 4-hydroxythalidomide (150 mg, 0.55 mmol) in DMF (4.0 mL), potassium carbonate (75 mg, 0.55 mmol) was added followed by tert-butyl (2-bromoethyl)carbamate (112 mg, 0.500 mmol). The solution was stirred for 16 h at room temperature. The crude mixture was partitioned between water (5.0 mL) and ethyl acetate (5.0 mL). The aqueous layer was further extracted with ethyl acetate (5.0 mL). The combined organic layer was washed with water (3 × 4 mL), then dried over Na_2SO_4, filtered, and concentrated *in vacuo*. The residue recovered was purified by silica gel flash chromatography (2–8% methanol in DCM) to afford **5** as a pale yellow oil (96 mg, 42%, containing 20% of bis alkylated material).**^{1}H NMR** (400 MHz, $CDCl_3$) δ 8.27 (s, 1H), 7.72 – 7.63 (m, 1H), 7.51 – 7.43 (m, 1H), 7.27 – 7.19 (m, 1H), 5.29 (s, 1H), 5.01 – 4.89 (m, 1H), 4.26 – 4.19 (m, 2H), 3.64 – 3.56 (m, 2H), 3.01 – 2.68 (m, 3H), 2.19 – 2.05 (m, 1H), 1.43 (s, 9H). **^{13}C NMR** (101 MHz, $CDCl_3$) δ 169.15, 168.22, 167.04, 165.92, 156.38, 136.83, 133.85, 119.39, 117.53, 116.47, 79.84, 79.40, 49.28, 40.50, 39.95, 31.95, 22.75, 22.19. **MS** (ESI) *m/z* 418 (M+H)$^+$.

(9H-fluoren-9-yl)methyl (E)-(4-((1-(2-((2-((2-(2,6-dioxopiperidin-3-yl)-1,3-dioxoisoindolin-4-yl)oxy)ethyl)amino)-2-oxoethyl)-3,5-dimethyl-1H-pyrazol-4-yl)diazenyl)benzyl)carbamate (6)

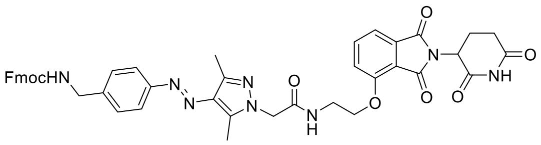

A solution of thalidomide **5** (96 mg, 0.230 mmol) in DCM (5.0 ml) was added HCl 4 M in Dioxane (0.57 mL, 2.30 mmol). The solution was stirred at room temperature for 4 h, then concentrated *in vacuo* to give a white powder which was used in the subsequent step without further purification. A solution of azopyrazole carboxylic acid **4** (50 mg, 0.09 mmol) in DCM (3.0 mL) were added HATU (102 mg, 0.268 mmol) and DIPEA (0.450 mmol, 78 µl). The crude deprotected thalidomide derivative (HCl salt, 32 mg, 0.090 mmol) was then added and the solution was stirred at room temperature for 16 h. The reaction mixture was subsequently washed twice with water (2 × 3 mL). The organic layer was dried over Na_2SO_4, filtered, and concentrated *in vacuo*. The residue recovered was purified by silica gel flash chromatography (2–8% methanol in DCM) to afford compound **6** as a yellow solid (29 mg, 33%). **^{1}H NMR** (400 MHz, $CDCl_3$) δ 8.28 (s, 1H), 7.79 – 7.71 (m, 4H), 7.68 – 7.56 (m, 4H), 7.48 – 7.43 (m, 1H), 7.42 – 7.38 (m, 2H), 7.36 – 7.27 (m, 4H), 7.19 – 7.13 (m, 1H), 4.81 – 4.73 (m, 2H), 4.72 – 4.61 (m, 1H), 4.52 – 4.38 (m, 4H), 4.28 – 4.16 (m, 3H), 3.89 – 3.77 (m, 1H), 3.75 – 3.60 (m, 1H), 2.70 – 2.59 (m, 1H), 2.55 (s, 3H), 2.47 (s, 3H), 2.45 – 2.39 (m, 1H), 2.39 – 2.26 (m, 1H), 2.08 – 1.90 (m, 2H). **^{13}C NMR** (101 MHz, $CDCl_3$) δ171.3, 167.4, 156.7, 156.6, 144.4, 144.0, 141.5, 140.4, 136.8, 133.8, 128.4, 128.3, 128.1, 127.8, 127.2, 125.2, 125.1, 122.2, 120.1, 119.6, 116.8, 68.1, 66.8, 53.5, 52.2, 47.4, 44.9, 38.8, 31.2, 22.6, 14.2, 10.0. **HRMS** (ESI) m/z: calculated for $C_{44}H_{41}N_8O_8^+$, 809.3047; found 809.3051.

2-((S)-4-(4-chlorophenyl)-2,3,9-trimethyl-6H-thieno[3,2-f][1,2,4]triazolo[4,3-a][1,4]diazepin-6-yl)-N-(4-((E)-(1-(2-((2-((2-(2,6-dioxopiperidin-3-yl)-1,3-dioxoisoindolin-4-yl)oxy)ethyl)amino)-2-oxoethyl)-3,5-dimethyl-1H-pyrazol-4-yl)diazenyl)benzyl)acetamide (AP-PROTAC-1)

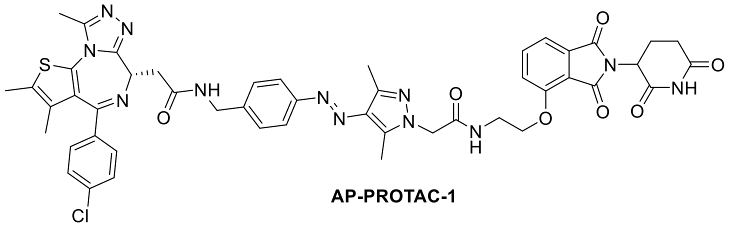

AP-PROTAC-1

A solution of compound **6** (25 mg, 0.03 mmol) in DMF (3 mL) was added piperidine (3 µL, 0.03 mmol) and the mixture was stirred at room temperature for 5 h. HATU (15 mg, 0.039 mmol) and DIPEA (0.06 mmol, 10 µL) were added, followed by JQ1-COOH (12 mg, 0.03 mmol) and the solution was stirred at room temperature for 16 h. The reaction mixture was washed with water (2 x 3 mL). The organic layer was dried over sodium sulfate, filtered, and concentrated *in vacuo*. The residue was purified by preparative HPLC (20–98% acetonitrile in water with 0.1% formic acid) to afford **AP-PROTAC-1** as a yellow solid (8 mg, 27%). **^{1}H NMR** (500 MHz, DMSO-d_6) δ 11.12 (s, 1H), 8.84 (t, J = 6.1 Hz, 1H), 8.51 – 8.47 (m, 1H), 7.87 – 7.77 (m, 1H), 7.73 – 7.66 (m, 2H), 7.60 – 7.54 (m, 1H), 7.53 – 7.44 (m, 3H), 7.44 – 7.39 (m, 2H), 7.37 – 7.31 (m, 2H), 5.13 – 5.05 (m, 1H), 4.81 (s, 2H), 4.58 – 4.45 (m, 3H), 4.38 – 4.24 (m, 3H), 3.57 – 3.50 (m, 1H), 3.43 – 3.36 (m, 1H), 3.27 – 3.19 (m, 1H), 2.94 – 2.83 (m, 1H), 2.60 (s, 3H), 2.63 – 2.56 (m, 1H), 2.59 – 2.51 (m, 1H), 2.52 (s, 3H), 2.40 (s, 3H), 2.37 (s, 3H), 2.07 – 1.99 (m, 1H), 1.60 (s, 3H).**^{13}C NMR** (126 MHz, DMSO-d_6) δ 172.8, 169.7, 166.8, 165.2, 163.1, 155.6, 155.1, 151.9, 149.9, 137.1, 136.7, 133.3, 132.3, 130.7, 130.2, 129.8, 129.6, 128.4, 128.1, 121.3, 67.4, 54.0, 48.8, 41.8, 37.7, 31.0, 14.1, 13.9, 12.7, 11.3, 9.5. **HRMS** (ESI) m/z: calculated for $C_{48}H_{46}N_{12}O_7SCl^+$, 969.3022; found 969.3033.

4-((2-(2,6-dioxopiperidin-3-yl)-1-oxoisoindolin-4-yl)amino)-4-oxobutanoic acid (7)

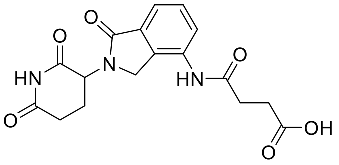

To a solution of lenalidomide (100 mg, 0.386 mmol) in DMF (4 mL), succinic anhydride (57.9 mg, 0.579 mmol) was added. The solution was stirred at 60 ˚C for 24 h, then added Et_2O (30 mL) and stored at 0 ˚C overnight for 18 h. A white precipitate was observed and collected with gravity filtration to yield carboxylic acid **7** as a white solid (138 mg, 99%). **^{1}H NMR** (400 MHz, DMSO-d_6) δ 12.18 (s, 1H), 11.04 (s, 1H), 9.88 (s, 1H), 7.87 – 7.77 (m, 1H), 7.55 – 7.45 (m, 2H), 5.16 (dd, J = 13.3, 5.1 Hz, 1H), 4.43 – 4.27 (m, 2H), 3.00 – 2.90 (m, 1H), 2.66 – 2.58 (m, 3H), 2.58 – 2.52 (m, 2H), 2.43 – 2.26 (m, 1H), 2.09 – 1.98 (m, 1H). **^{13}C NMR** (101 MHz, DMSO-d_6) δ 174.3, 173.4, 171.6, 170.8, 168.3, 134.2, 134.1, 133.1, 129.1, 125.5, 119.4, 52.0, 46.9, 31.7, 31.1, 29.4, 23.1. **HRMS** (ESI) m/z: calculated for $C_{17}H_{18}N_3O_6^+$, 360.1195; found 360.1194.

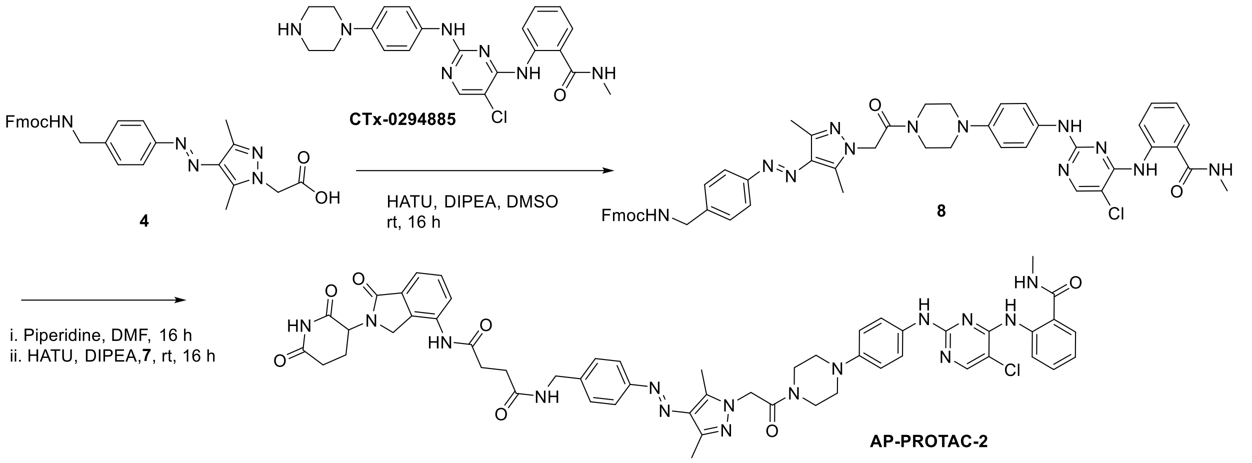

Scheme S2. Synthesis of kinase **AP-PROTAC-2**

(E)-N1-(4-((1-(2-(4-(4-((5-chloro-4-((2-(methylcarbamoyl)phenyl)amino)pyrimidin-2-yl)amino)phenyl)piperazin-1-yl)-2-oxoethyl)-3,5-dimethyl-1H-pyrazol-4-yl)diazenyl)benzyl)-N4-(2-(2,6-dioxopiperidin-3-yl)-1-oxoisoindolin-4-yl)succinimide (AP-PROTAC-2)

Carboxylic acid **4** (90 mg, 0.176 mmol) was dissolved in a mixture of DMSO (2 mL) and DMF (2 mL). Amine **CTx0294885** (77 mg, 0.176 mmol), HATU (100 mg, 0.264 mmol) and DIPEA (0.122 mL, 0.704 mmol) were added to the mixture. The mixture was stirred at room temperature for 16 h, then added water (20 mL) and extracted with ethyl acetate (5 × 20 mL). The combined organic layer was washed with brine, dried over Na_2SO_4 and concentrated *in vacuo*. The crude was purified by flash column chromatography (2%–8% methanol in DCM) to afford intermediate **8** as an orange solid (100 mg) which was used in the subsequent step without further characterisation.

To a solution of intermediate **8** (100 mg, 0.108 mmol) in DMF (2 mL), piperidine (0.011 mL, 0.108 mmol) was added. The reaction mixture was stirred at room temperature for 16 h. HATU (62 mg, 0.162 mmol), DIPEA (0.075 mL, 0.149 mmol) and lenalidomide derivative **7** (39 mg, 0.108 mmol) were added into the reaction mixture. The mixture was stirred at room temperature for 16 h. The crude mixture was added water (20 mL) and then extracted with ethyl acetate (5 × 20 mL). The combined organic layer was washed with brine and dried over Na_2SO_4, filtered, concentrated *in vacuo* and freeze-dried with water. The crude residue was purified by flash column chromatography (3%–7% methanol in DCM) to give desired product **AP-PROTAC-2** as a yellow solid (12.5 mg, 6.8% two steps). **^{1}H NMR** (400 MHz, DMSO-d_6) δ 11.61 (s, 1H), 11.03 (s, 1H), 9.27 (s, 1H), 8.80 – 8.75 (m, 2H), 8.55 – 8.49 (m, 1H), 8.17 (s, 1H), 7.90 – 7.81 (m, 1H), 7.79 – 7.72 (m, 1H), 7.70 – 7.62 (m, 2H), 7.56 – 7.42 (m, 5H), 7.42 – 7.35 (m, 2H), 7.27 – 7.23 (m, 1H), 7.18 – 7.08 (m, 1H), 7.00 – 6.89 (m, 2H), 5.22 (s, 2H), 5.18 – 5.03 (m, 1H), 4.43 – 4.24 (m, 4H), 3.73 – 3.60 (m, 4H), 3.21 – 3.16 (m, 2H), 3.13 – 3.06 (m, 2H), 2.98 – 2.84 (m, 1H), 2.81 (d, *J* = 4.5 Hz, 3H), 2.71 – 2.52 (m, 5H), 2.48 (s, 3H), 2.37 (s, 3H), 2.34 – 2.23 (m, 1H), 2.05 – 1.96 (m, 1H). **^{13}C NMR** (101 MHz, DMSO-d_6) δ 172.9, 171.3, 171.1, 170.7, 168.9, 167.8, 164.7, 158.0, 154.9, 154.7, 152.0, 146.2, 141.2, 141.1, 140.5, 139.4, 134.5, 133.9, 133.5, 132.9, 132.7, 131.5, 128.6, 128.0, 127.9, 125.0, 121.8, 121.3, 121.2, 120.5, 118.9, 116.5, 104.4, 51.5, 50.5, 49.5, 49.1, 46.4, 44.2, 41.8, 41.6, 31.3, 31.2, 30.5, 26.3, 22.7, 13.9, 9.6. **MS** (MALDI-TOF) *m/z*: calculated for $C_{53}H_{55}ClN_{15}O_7^+$, 1048.4; found 1049.0.

Compound purity on LC-MS

Compound	Retention time (min)	Purity (area% for both isomers)	Method
**AP-PROTAC-1**	*Z*, 2.40; *E*, 2.73	94%	50% to 98% acetonitrile in water with 0.1% formic acid over 12 min.
**AP-PROTAC-2**	*Z*, 9.04; *E*, 9.84	97%	20% to 98% acetonitrile in water with 0.1% formic acid over 12 min.

NMR spectra

(9H-fluoren-9-yl)methyl (4-aminobenzyl)carbamate (1)

^{1}H NMR (400 MHz, DMSO-d_6)

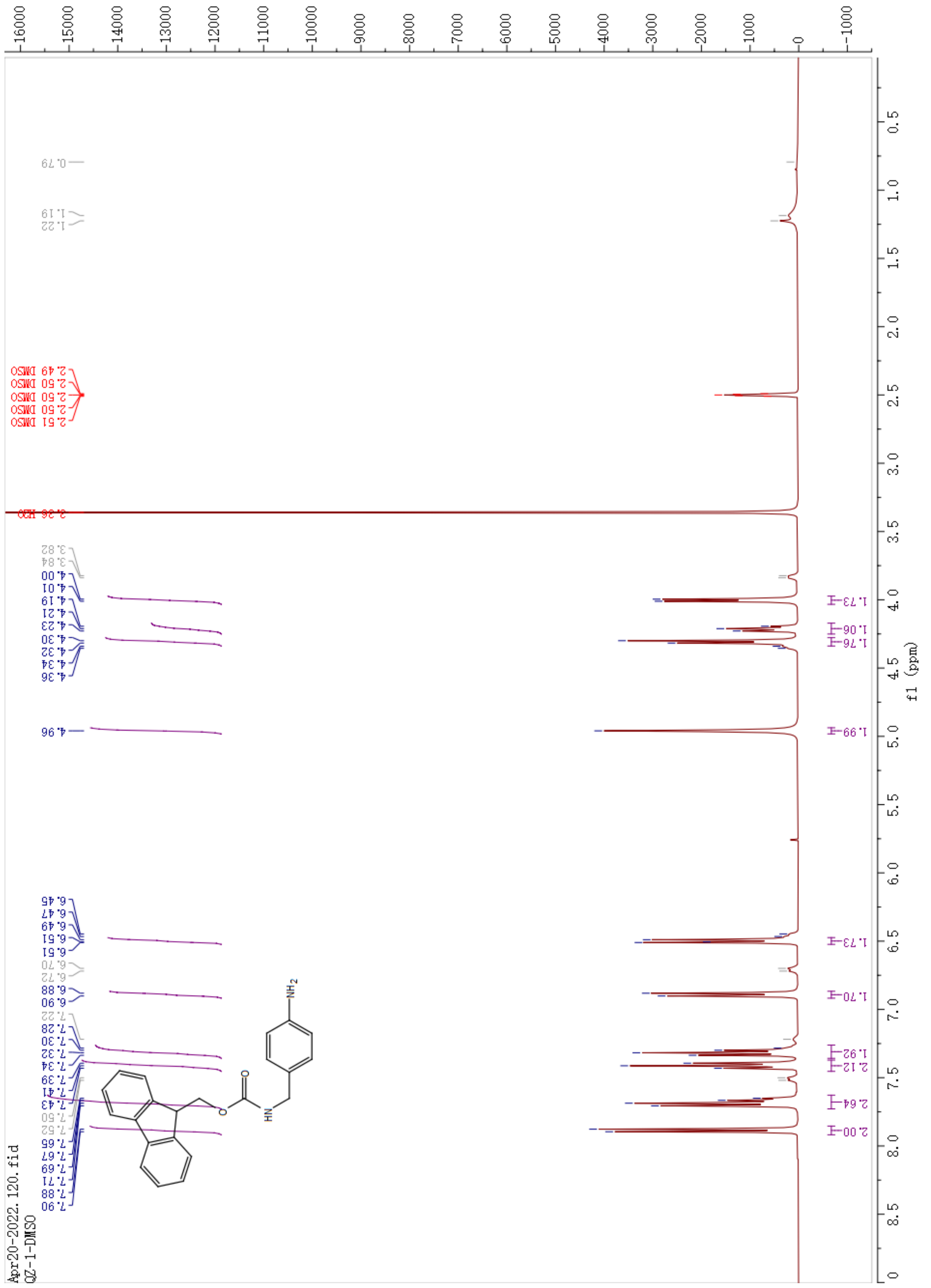

^{13}C NMR (101 MHz, DMSO-d_6)

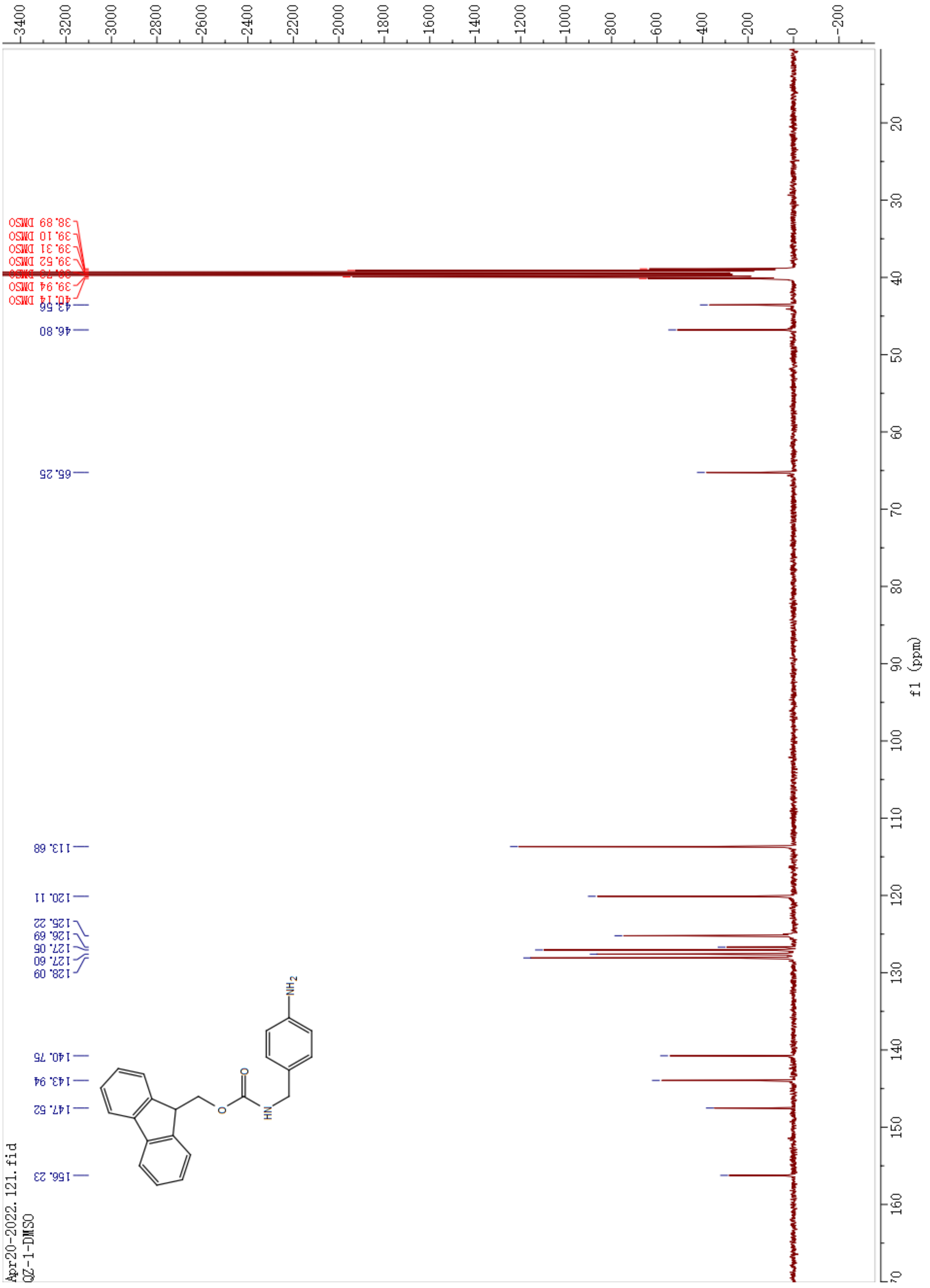

(9H-fluoren-9-yl)methyl(4-(2-(2,4-dioxopentan-3-ylidene)hydrazineyl)benzyl)carbamate (2)

^{1}H NMR (400 MHz, $CDCl_3$)

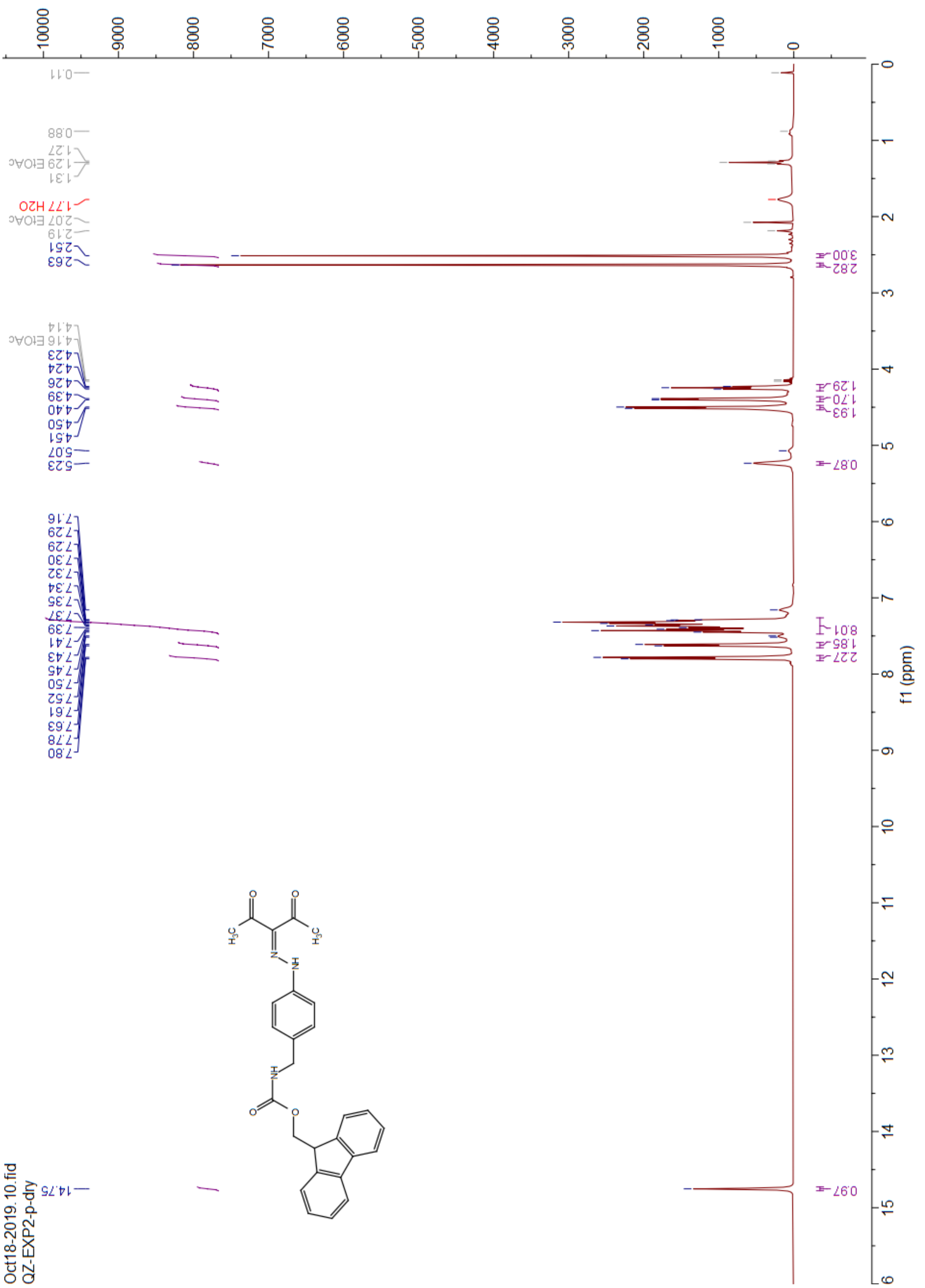

^{13}C NMR (101 MHz, $CDCl_3$)

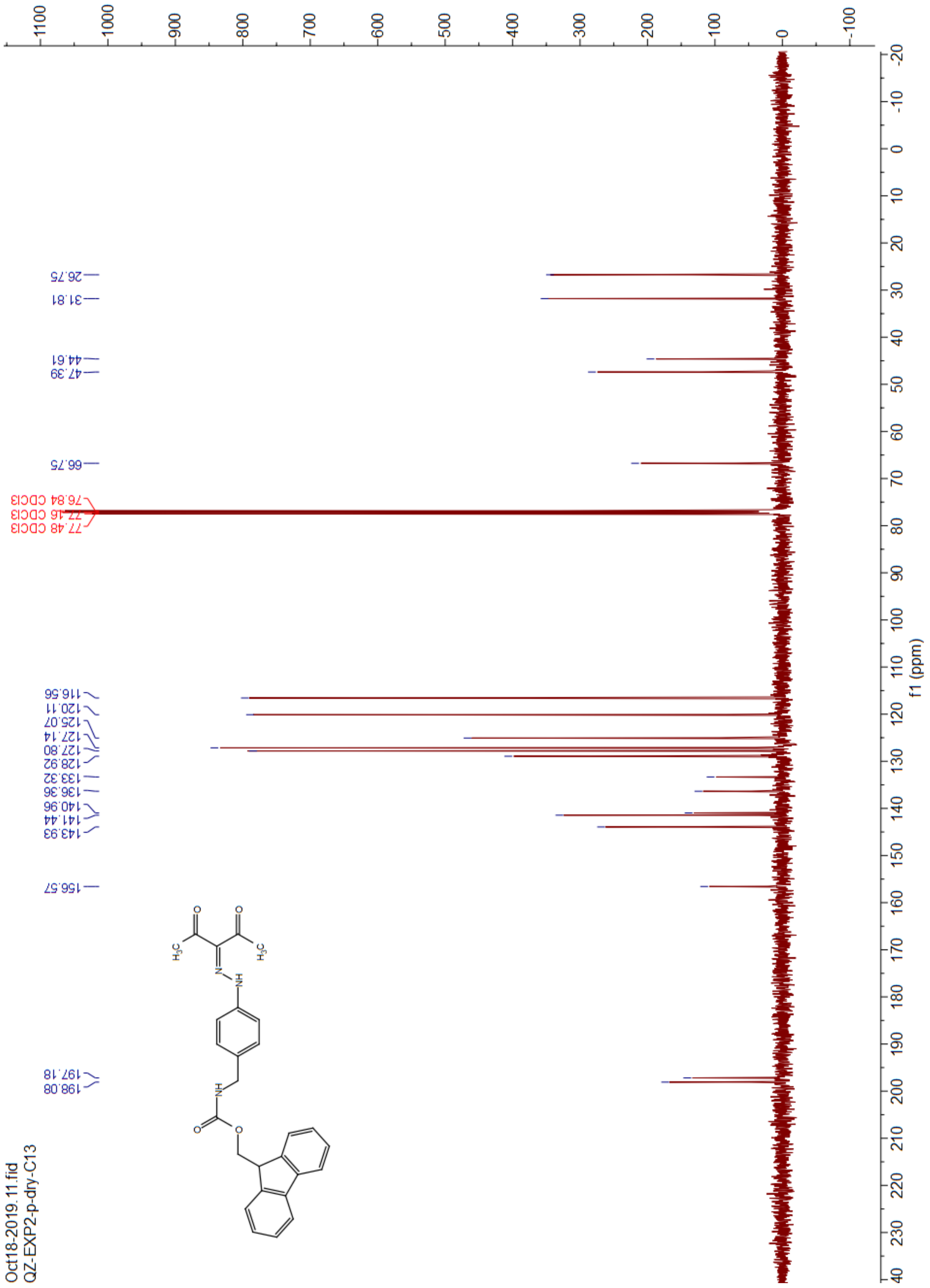

(9H-fluoren-9-yl)methyl(E)-(4-((1-(2-hydroxyethyl)-3,5-dimethyl-1H-pyrazol-4-yl)diazenyl)benzyl)carbamate (3)

^{1}H NMR (400 MHz, CDCl3)

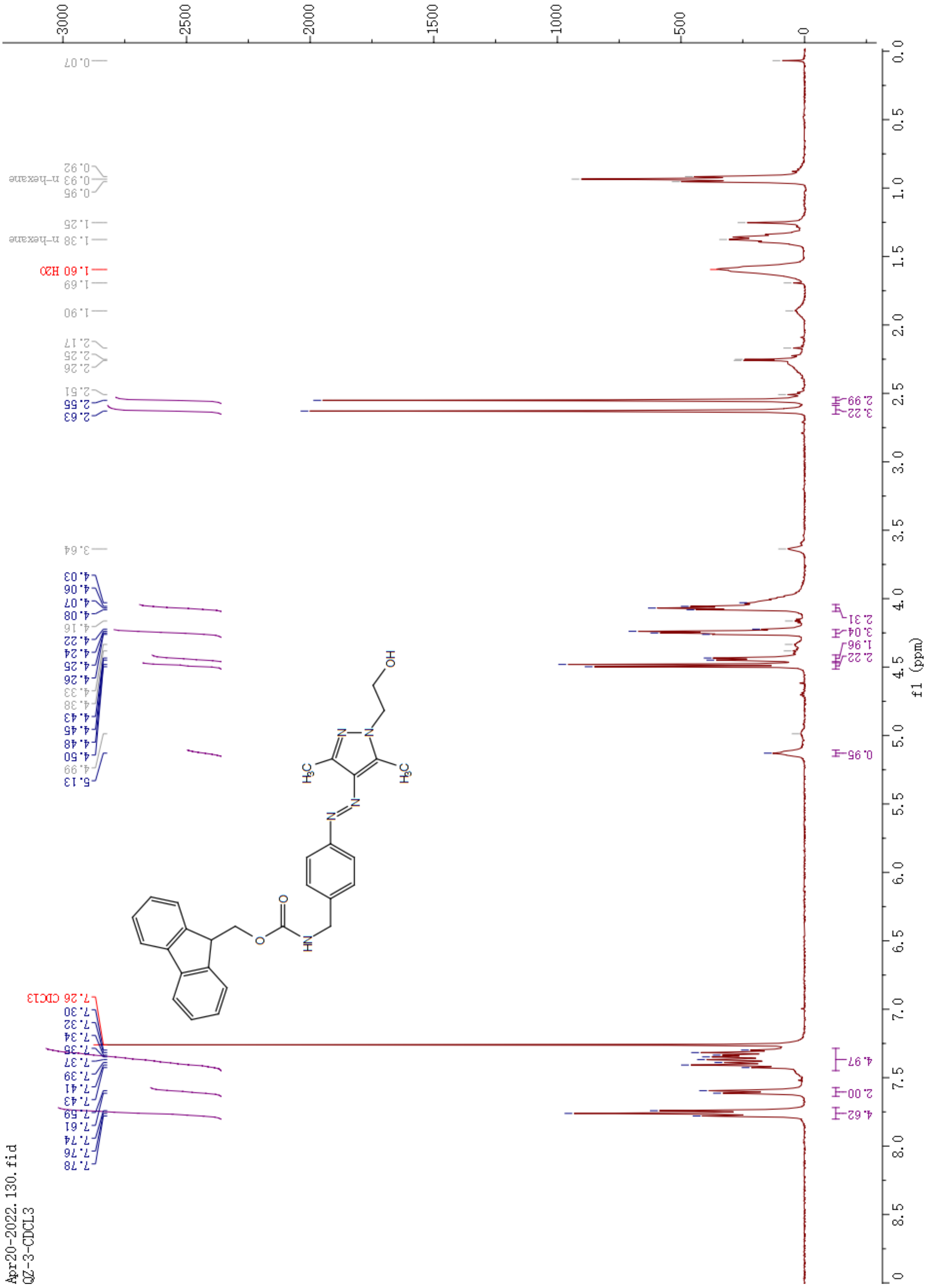

^{13}C NMR (101 MHz, $CDCl_3$)

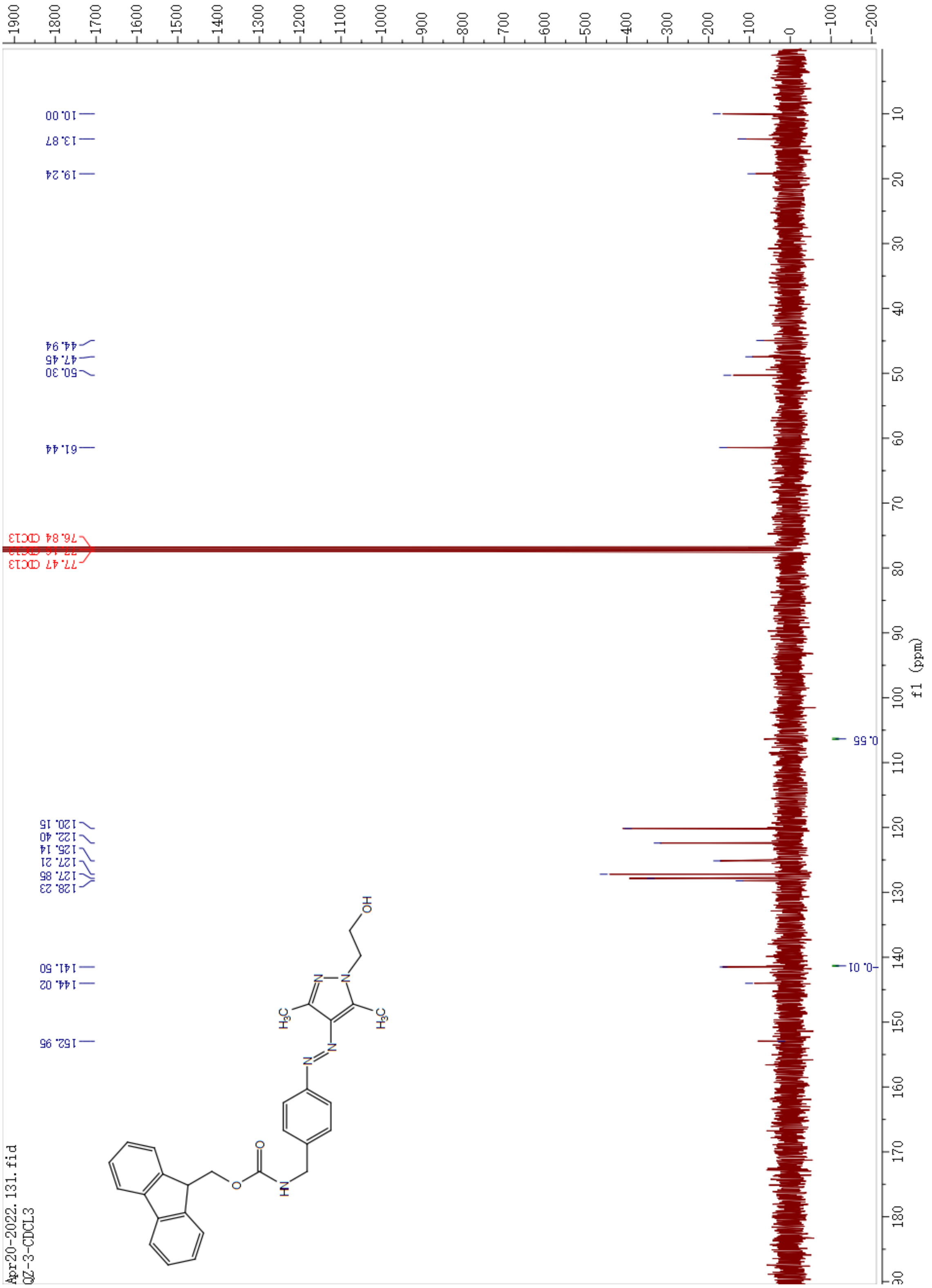

(E)-2-(4-((4-(((((9H-fluoren-9-yl)methoxy)carbonyl)amino) methyl)phenyl)diazenyl)-3,5-dimethyl-1H-pyrazol-1-yl) acetic acid (linker 4)

^{1}H NMR (400 MHz, DMSO-d_6), >99.9% *E* isomer

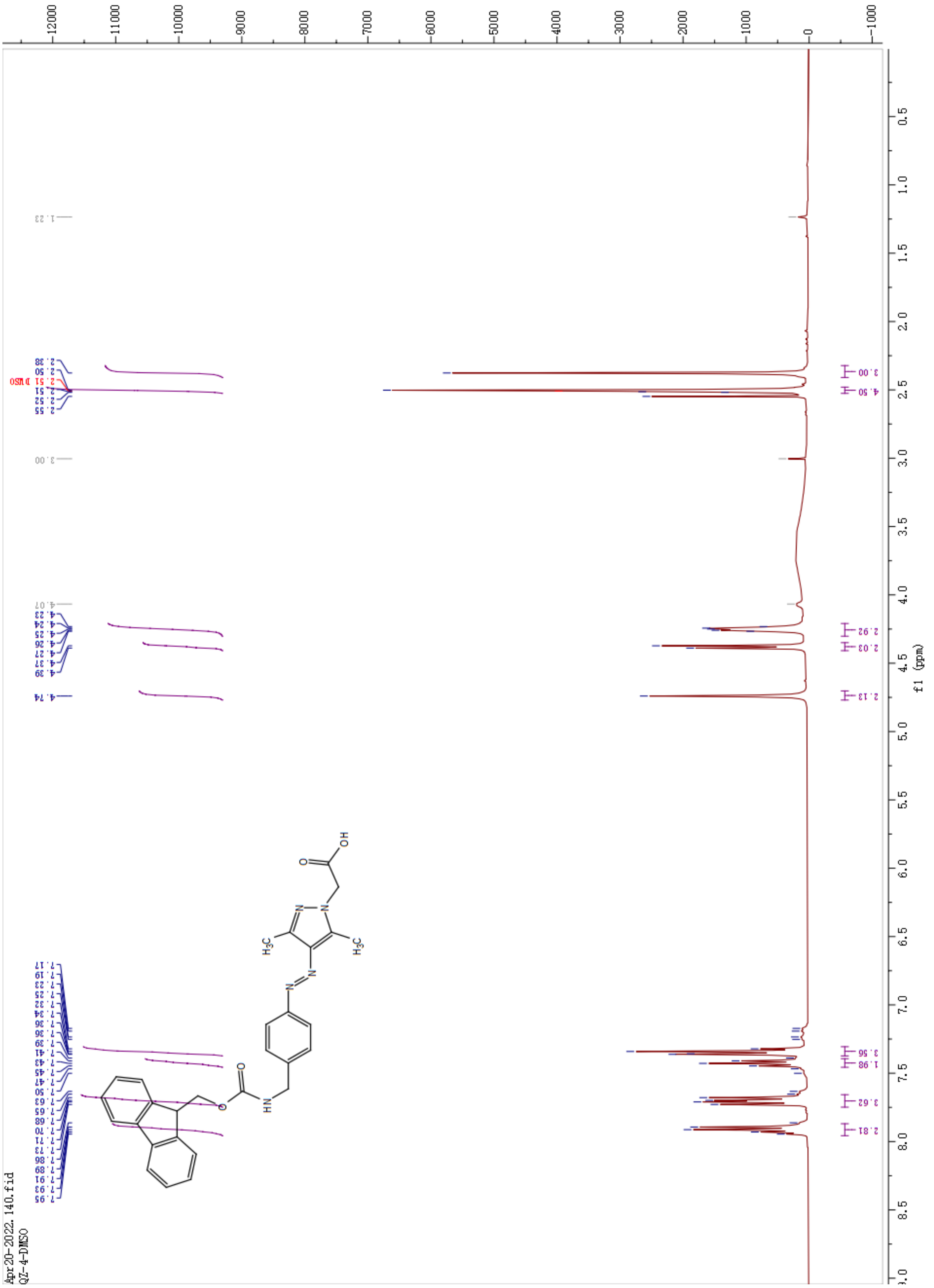

^{13}C NMR (101 MHz, DMSO-d_6)

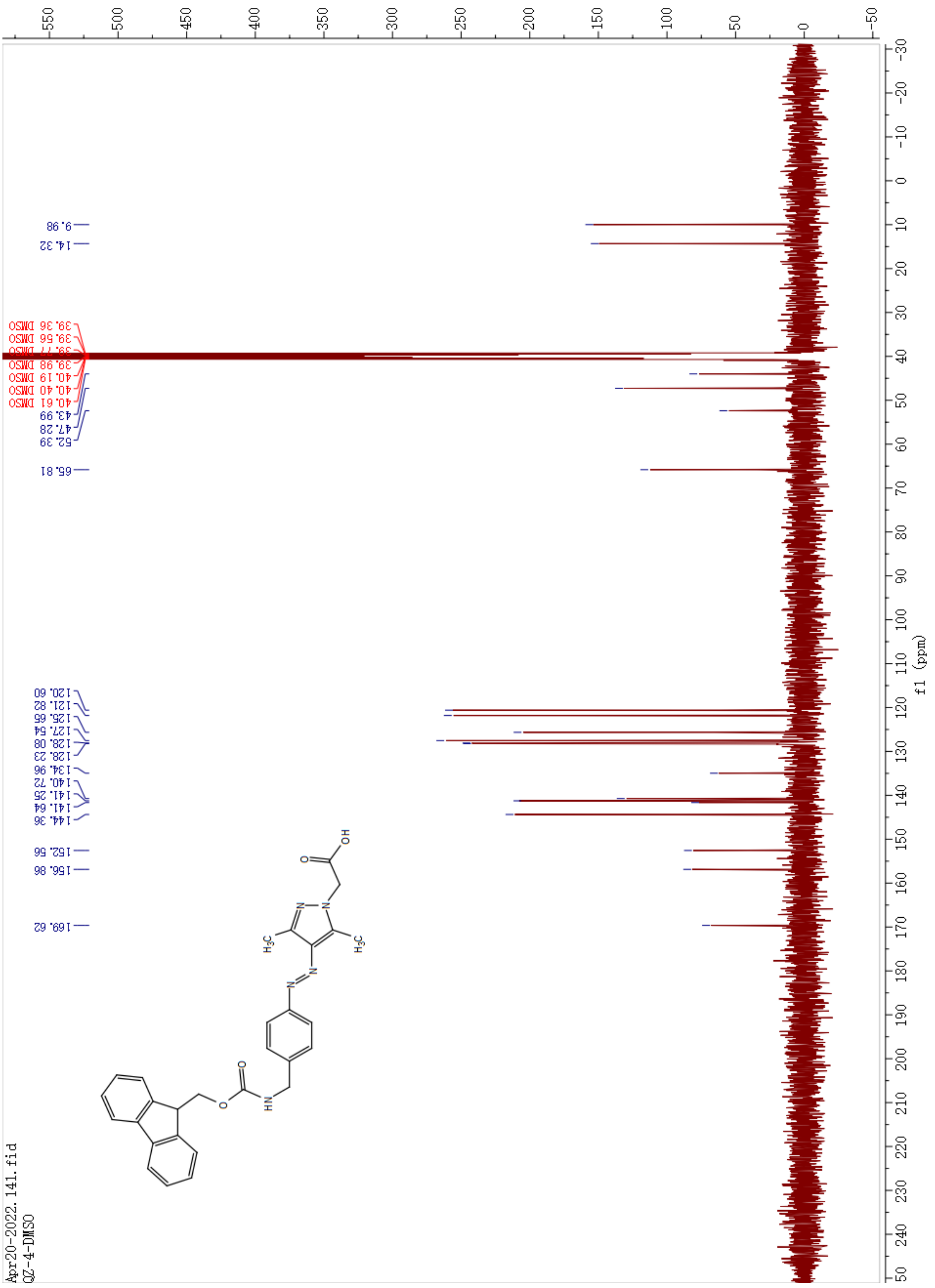

Tert-butyl (2-((2-(2,6-dioxopiperidin-3-yl)-1,3-dioxoisoindolin-4-yl)oxy)ethyl)carbamate (5)

^{1}H NMR (400 MHz, $CDCl_3$)

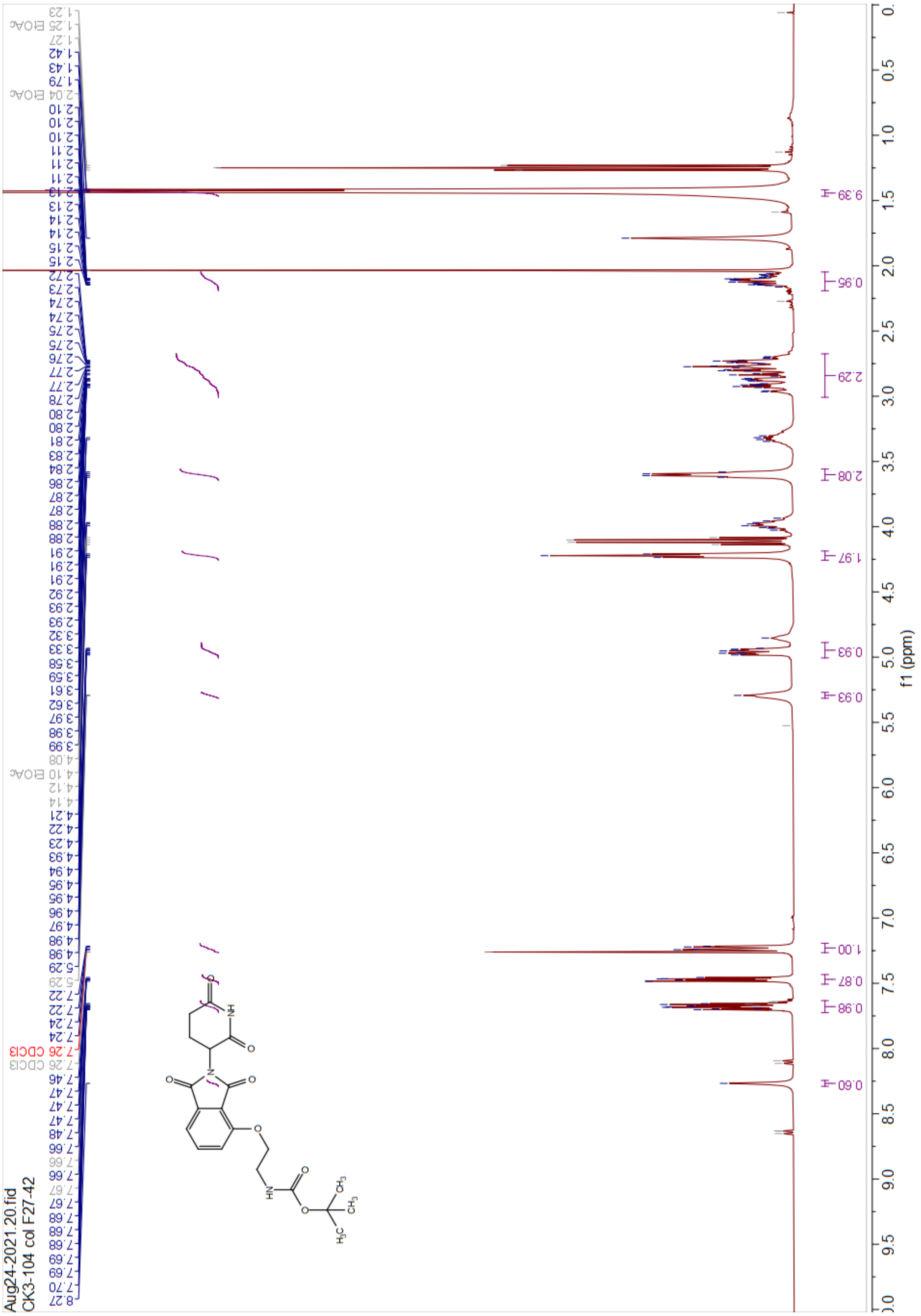

^{13}C NMR (101 MHz, $CDCl_3$)

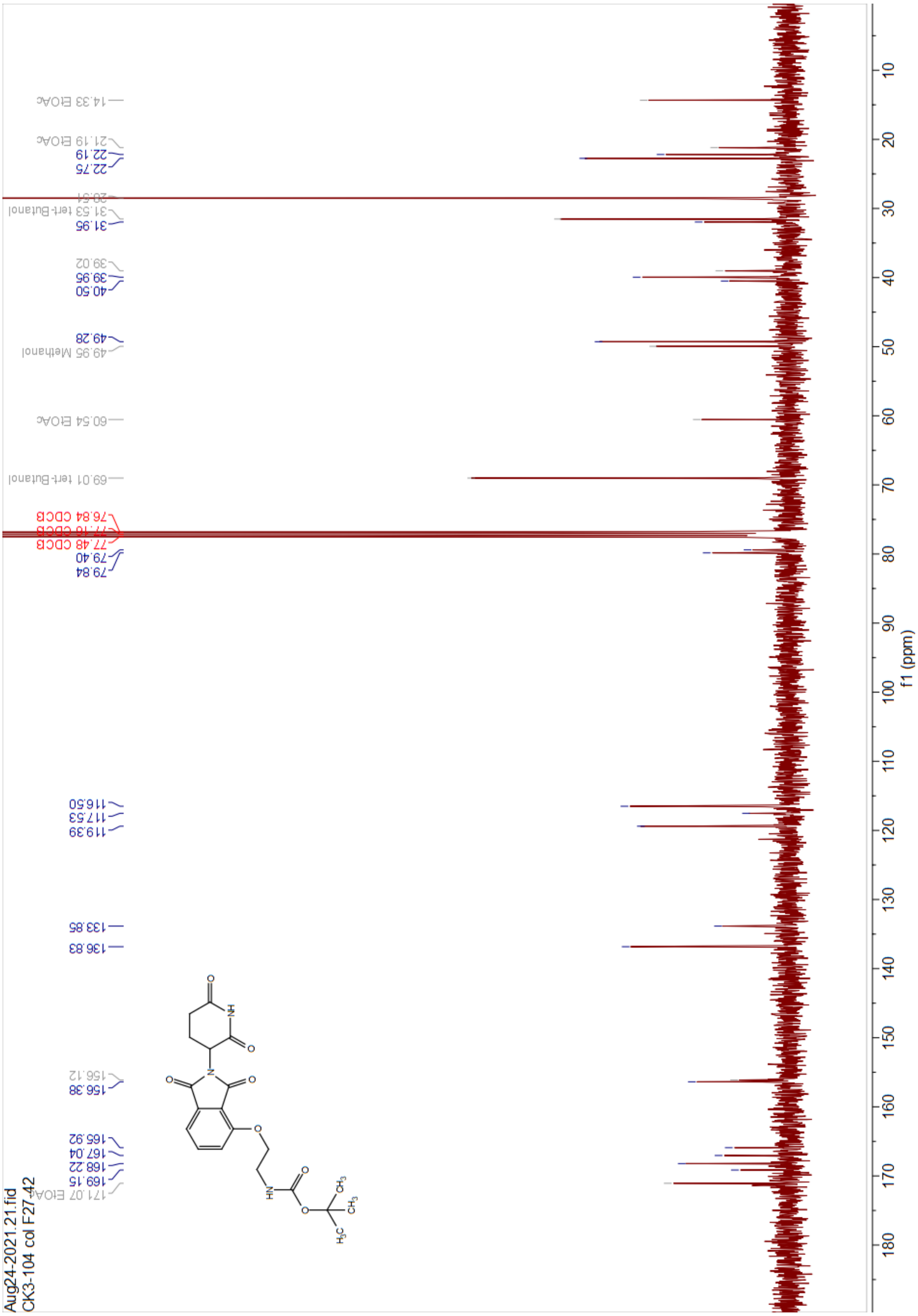

(9H-fluoren-9-yl)methyl (E)-(4-((1-(2-((2-((2-(2,6-dioxopiperidin-3-yl)-1,3-dioxoisoindolin-4-yl)oxy)ethyl)amino)-2-oxoethyl)-3,5-dimethyl-1H-pyrazol-4-yl)diazenyl)benzyl)carbamate (6)

^{1}H NMR (400 MHz, $CDCl_3$)

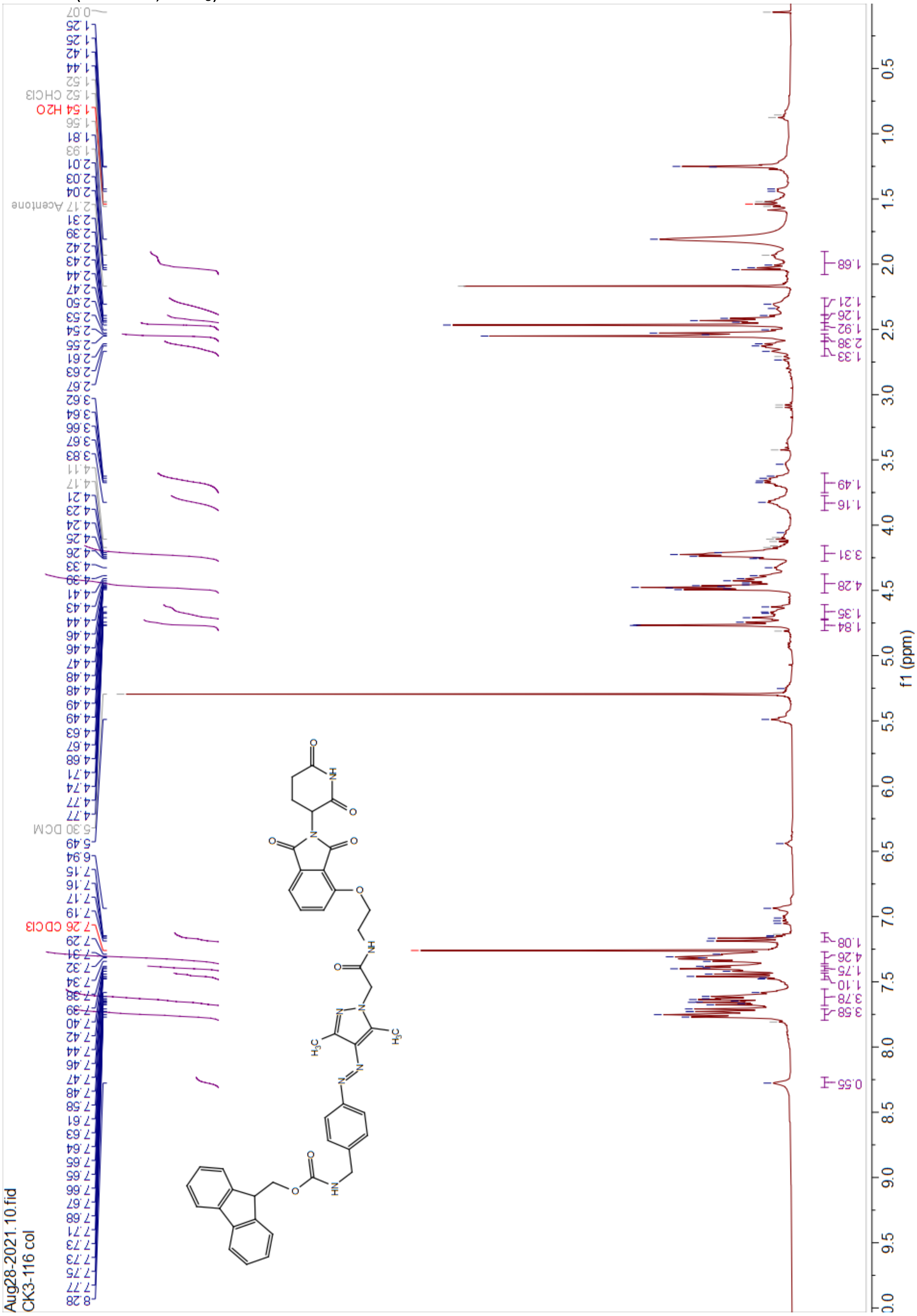

^{13}C NMR (101 MHz, $CDCl_3$)

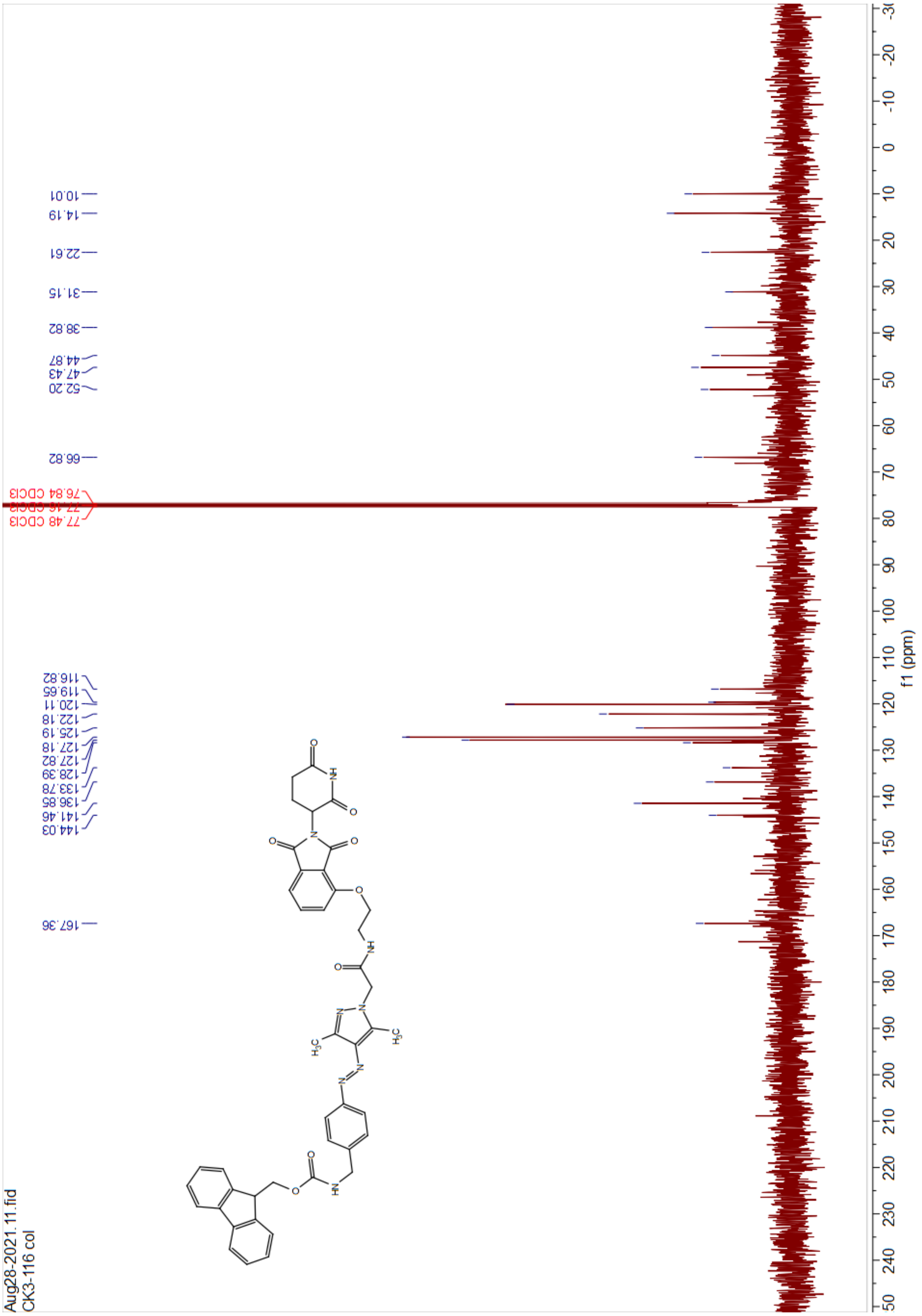

2-((S)-4-(4-chlorophenyl)-2,3,9-trimethyl-6H-thieno[3,2-f][1,2,4]triazolo[4,3-a][1,4]diazepin-6-yl)-N-(4-((E)-(1-(2-((2-((2-(2,6-dioxopiperidin-3-yl)-1,3-dioxoisoindolin-4-yl)oxy)ethyl)amino)-2-oxoethyl)-3,5-dimethyl-1H-pyrazol-4-yl)diazenyl)benzyl)acetamide (AP-PROTAC-1)

^{1}H NMR (500 MHz, DMSO-d_6)

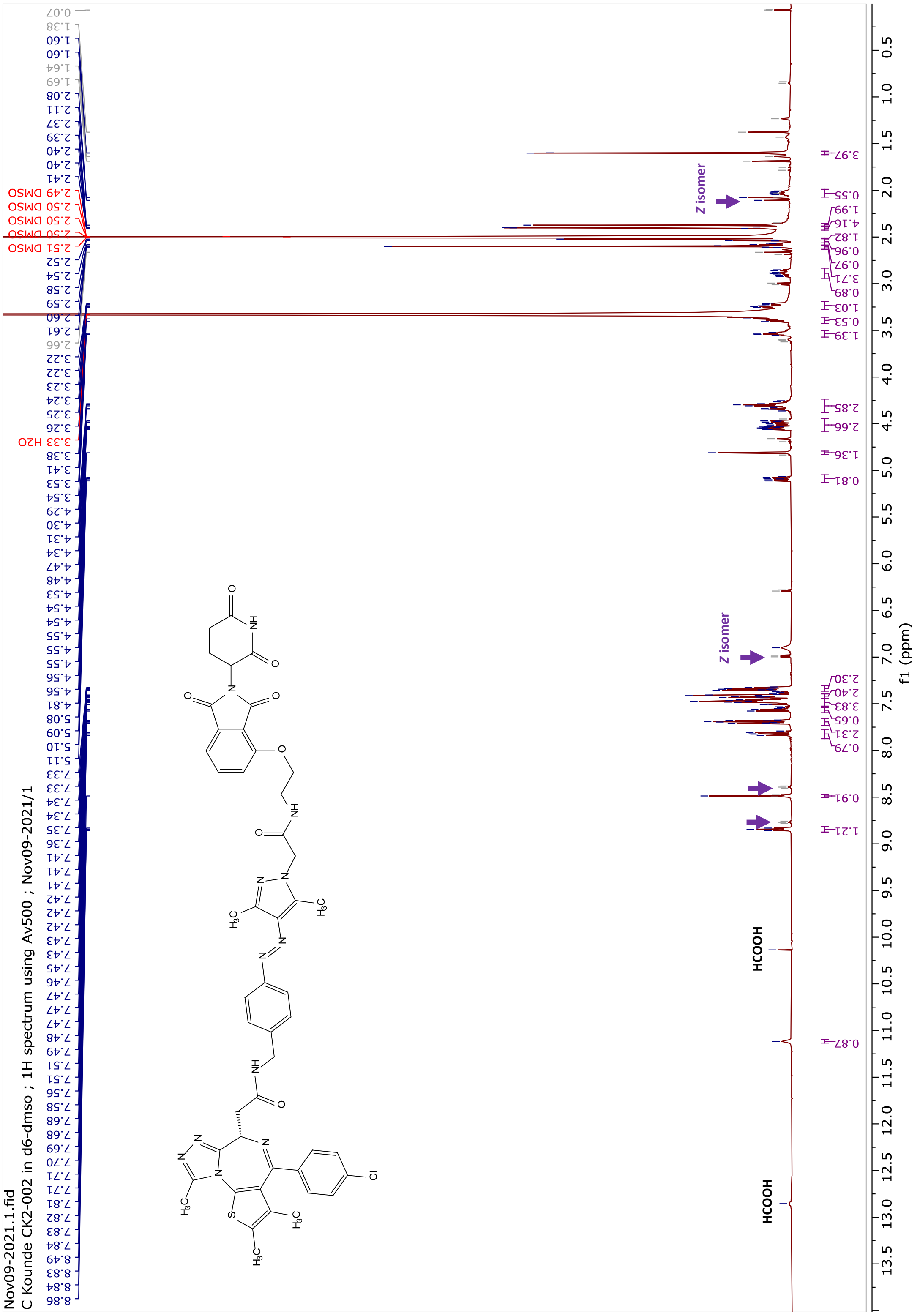

^{13}C NMR (126 MHz, DMSO-d_6)

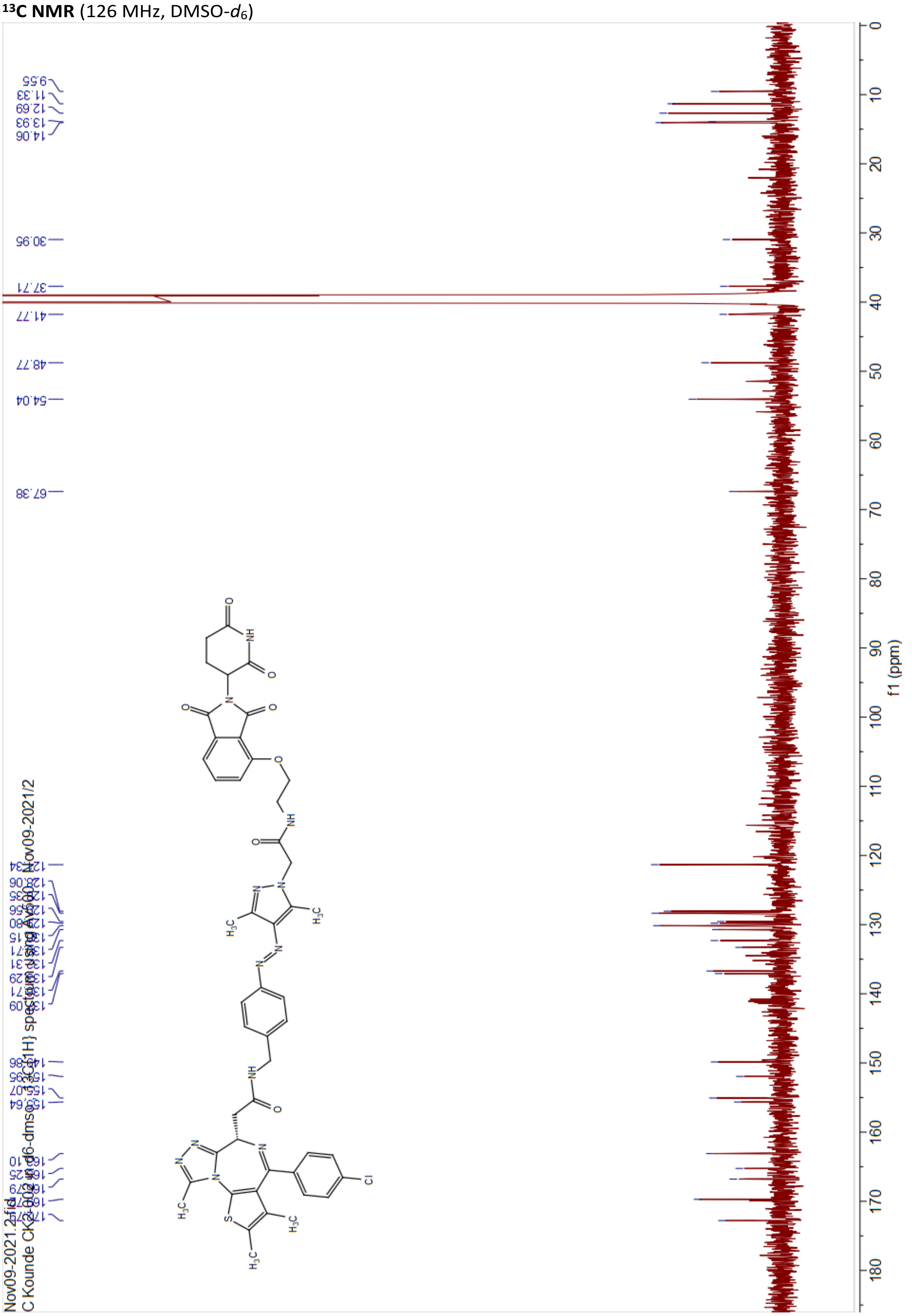

4-((2-(2,6-dioxopiperidin-3-yl)-1-oxoisoindolin-4-yl)amino)-4-oxobutanoic acid (7)

^{1}H NMR (400 MHz, DMSO-d_6)

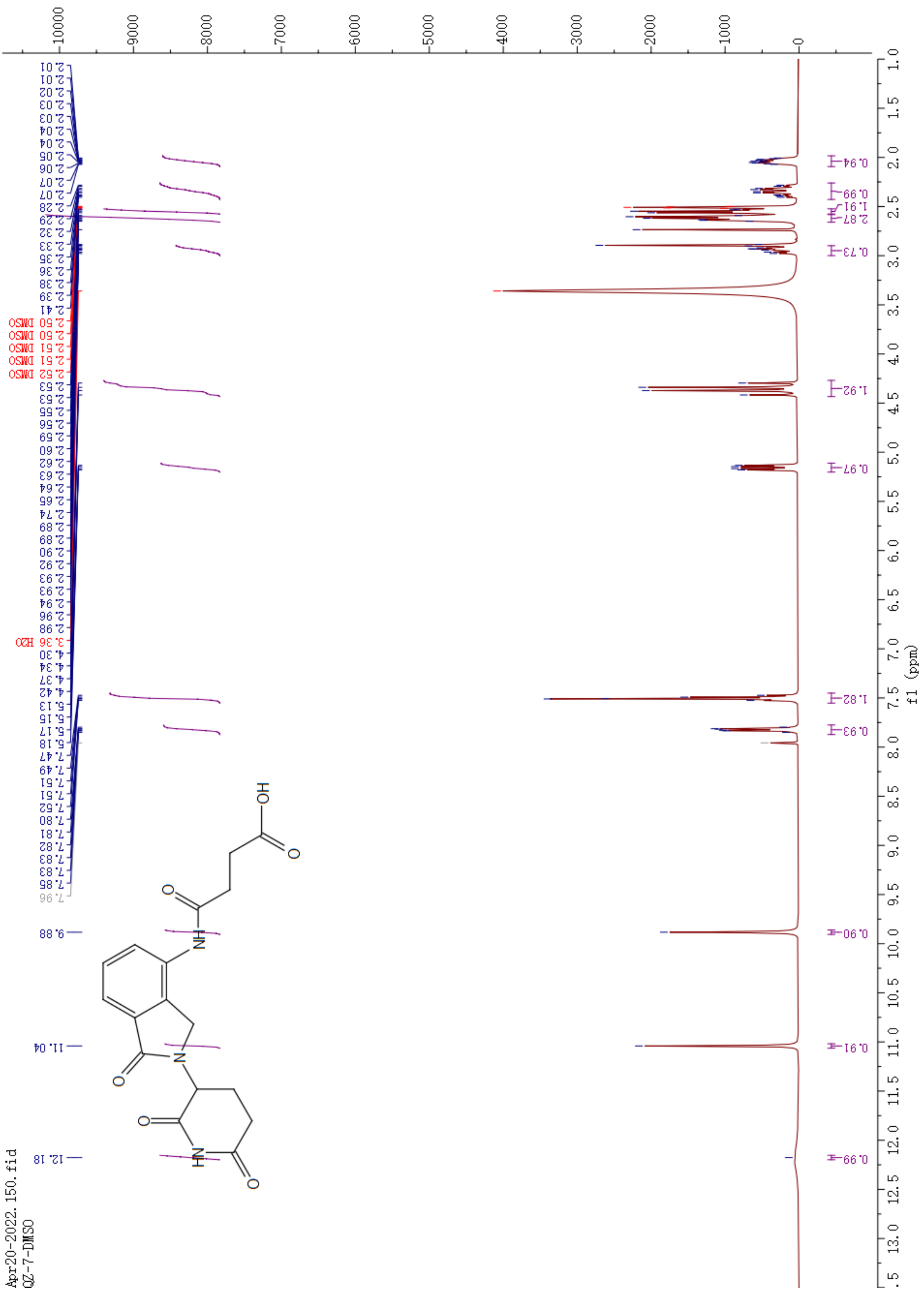

^{13}C NMR (101 MHz, DMSO-d_6)

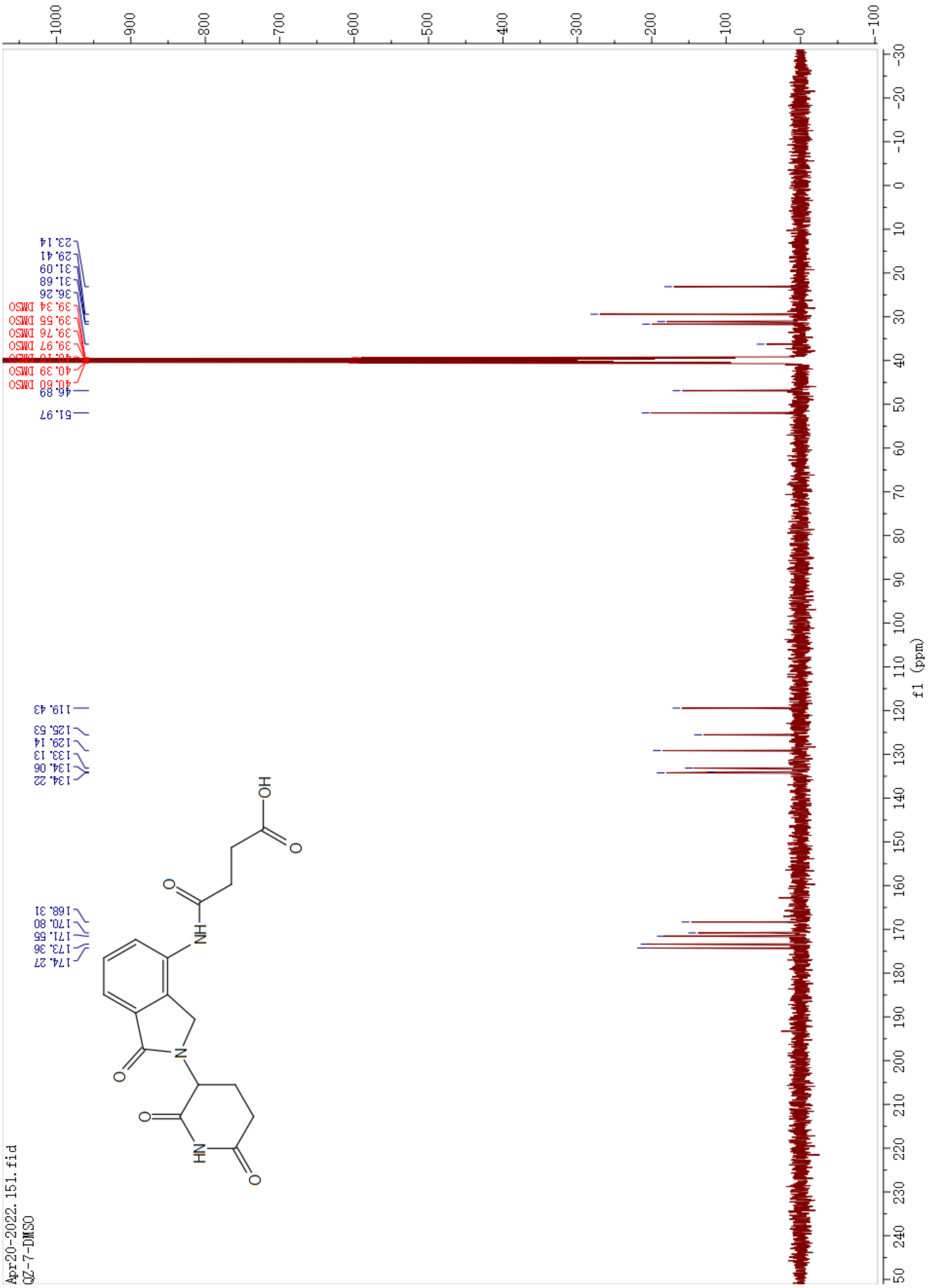

(E)*-N1-(4-((1-(2-(4-(4-((5-chloro-4-((2-(methylcarbamoyl)phenyl)amino)pyrimidin-2-yl)amino)phenyl)piperazin-1-yl)-2-oxoethyl)-3,5-dimethyl-1H-pyrazol-4-yl)diazenyl)benzyl)-N4-(2-(2,6-dioxopiperidin-3-yl)-1-oxoisoindolin-4-yl)succinimide (AP-PROTAC-2)

^{1}H NMR (400 MHz, DMSO-d_6)

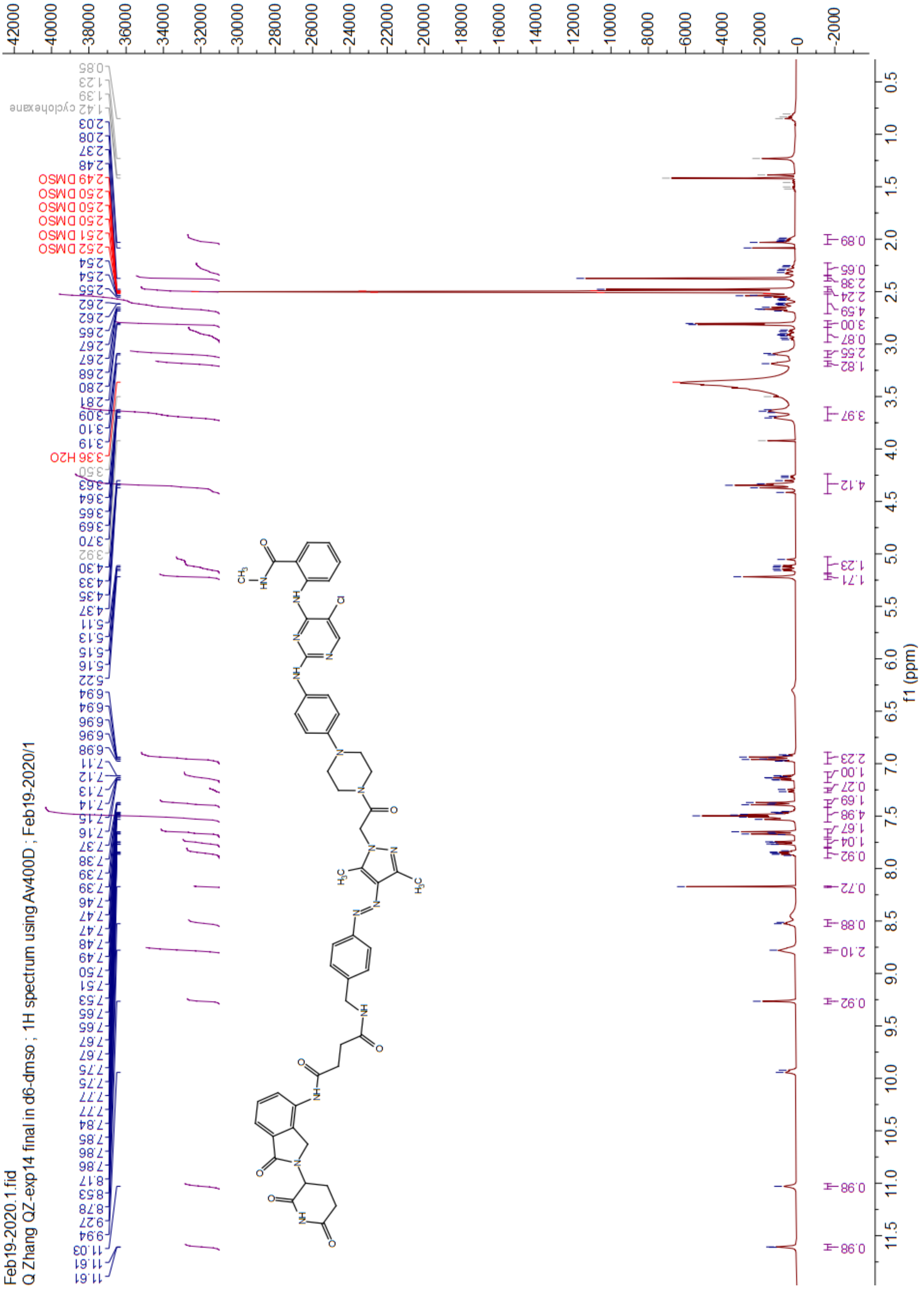

^{13}C NMR (101 MHz, DMSO-*d*$_6$)

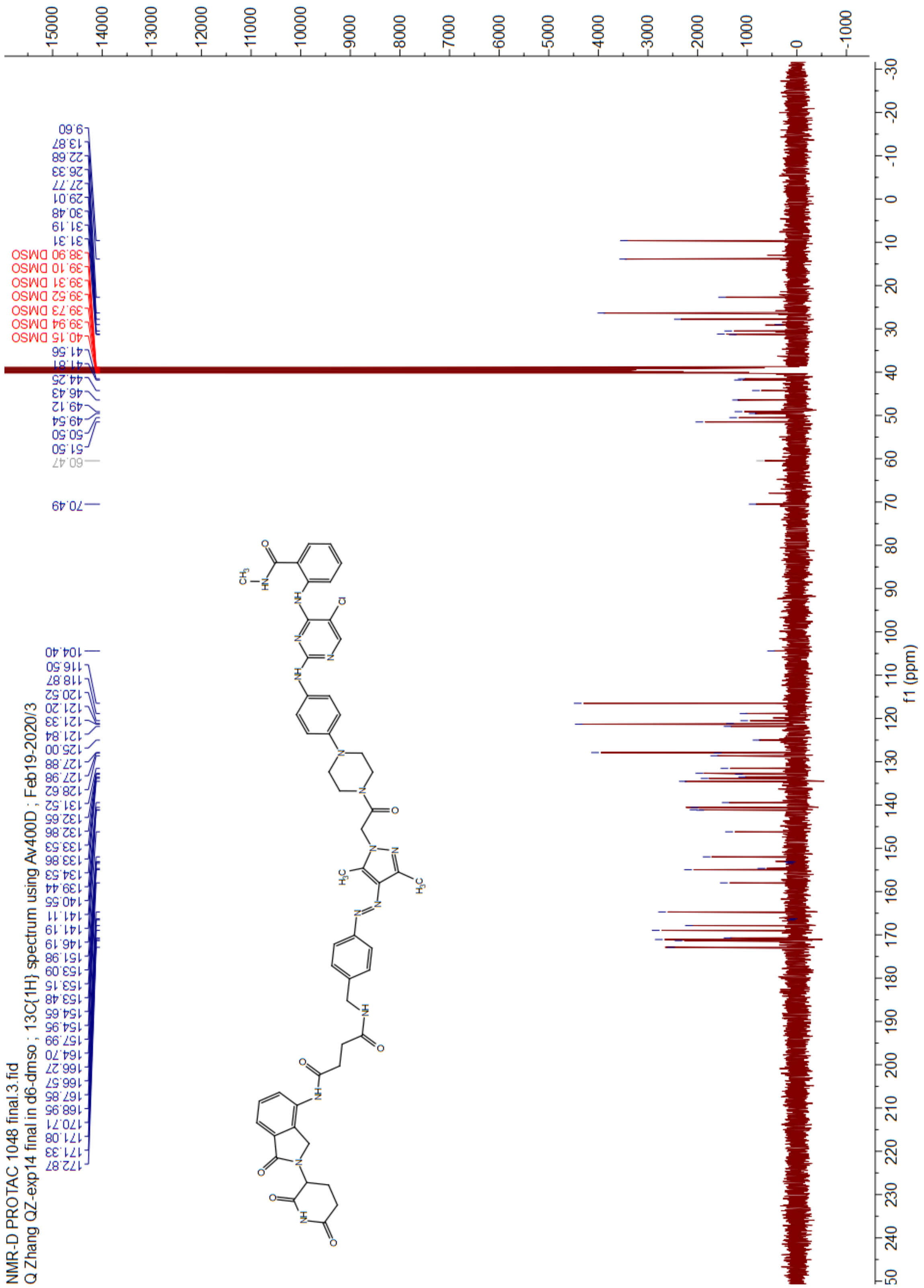

Supplementary Information: Biology

Cell culture and treatment

HeLa cells and MDA-MB-231 cells were obtained from the Francis Crick Institute cell banking. Cells were cultured in low-glucose Dulbecco's Modified Eagle's Medium (DMEM, Sigma, D6046) supplemented with 10% fetal bovine serum (FBS, Gibco) and grown in a humidified incubator at 37 °C, 5% CO_2. Cells were plated at 3 × 10^5 cells per well in a 6-well plate, allowed to adhere for 24 h before being treated with the indicated compounds and time, with 1 min irradiation of the cell flask at either 365 nm or 457 nm using LED light every 2 to 3 hours.

Cell Lysis

After cell treatment, cells were washed twice with PBS, added the respective cold lysis buffer and collected into eppendorf. Cells were lysed on ice for 30 min. The collected suspensions were centrifuged for 10 min at 17,000 g, 4 °C, and then the supernatant was collected. The protein concentration of each sample was determined using the DC™ Protein assay kit (BIO-RAD) with a bovine serum albumin standard curve in lysis buffer and normalized. The lysates were used in the subsequent immunoblotting, and proteomics sample preparation steps, or stored at −80 °C.

HeLa cells were lysed with RIPA buffer (75 mM NaCl, 0.25% sodium deoxycholate, 0.5% Triton X-100, 0.05% SDS and 50 mM Tris in Milli-Q purified water, pH 7.4). Benzonase (1 µL of 250 units/µL, E1014-25KU) and protease inhibitor (cOmplete, Roche) were added fresh to 10 mL of lysis buffer. MDA-MD-231 cells were lysed with PBS lysis buffer (1% Triton-X, 0.1% SDS, 1xPBS in Milli-Q purified water, pH 7.4) with protease inhibitor (cOmplete, EDTA-free) added fresh.

Immunoblotting

Lysates containing an equal amount of proteins (10 µg- 20 µg) were mixed 3:1 with 4x Laemmli protein sample buffer (supplemented with 10% 2-mercaptoethanol, BIO-RAD) and denatured at 95 °C for 10 min. Samples were resolved with Mini-PROTEAN® TGX™ Precast 7.5% gels (50 min, 160 V) and then wet transferred onto a nitrocellulose membrane. The membrane was blocked for 1 h at RT with 5% skimmed milk in Tris-buffered saline with 0.1 % Tween-20 (TBS-T). Primary antibodies were diluted in 5% milk in TBS-T and incubated with the membrane at 4 °C overnight. The membrane was washed with TBS-T (3 × 10 min), cut and incubated with appropriate IgG H&L HRP-conjugated secondary antibodies for 1 h at RT. The membrane was washed with TBST (3 × 10 min), and the bands were detected by chemiluminescence using the western HRP substrate (Merck Immobilon Crescendo). The images were recorded with ImageQuant Las 4000 and processed with ImageJ software. Primary antibodies used (1:1000 dilution): BRD4 (Cell Signalling Technology #13440), BRD2 (Cell Signalling Technology #5848), FAK (Cell Signalling Technology #3285), TBK1 (Cell Signalling Technology #3504), AURORA-A (Cell Signalling Technology #4718), GAK (Invitrogen #PA5-99201), β-actin (SIGMA-ALDRICH, A1978, 1:2500), GAPDH (Abcam, ab9485, 1:2500). Secondary antibodies used (1:10,000 dilution): Goat-α-mouse IgG (H+L) HRP conjugate (R-05071-500), Goat-α-rabbit IgG (H+L) HRP conjugate (R-05072-500).

Proteomics

Sample Preparation

The lysates of treated cells (100 µg, 100 µL) were reduced and alkylated by the addition of TCEP (to 5 mM final concentration) and CAA (to 15 mM final concentration) for 45 min at RT under vigorous shaking. Proteins were precipitated by adding 2 sample volumes of methanol, 0.5 volume of chloroform and 1 volume of purified water. The mixture was vortexed and centrifuged at 6,000 rpm for 2 min resulting in pellets of precipitated proteins. The pellets were washed with 3 sample volumes of methanol by vortexing and sonicating and then centrifuged at 8,000 rpm for 4 min. The supernatant was discarded, and the washing procedure was repeated an additional four times. The protein precipitate was air-dried for 5 min and redissolved in 100 µL of 50 mM HEPES pH 8.0 with sonication and vortexing. The protein solution was digested with trypsin protease (0.4 µg, 1:250 to protein, Pierce™ 90057) and incubated overnight in a thermoshaker with 800 rpm shaking at 37 °C. Trypsin digestion was quenched with the addition of 0.5% TFA. Peptide quantification assay (Pierce Fluorometric Peptide Assay) was performed. The same volume of peptide digest from each condition was aliquoted and dried with a SpeedVac Vacuum concentrator. The dried peptide samples were labelled with tandem mass tags (TMT10plex ref 90110, Thermo Scientific) for 2 h at RT. The TMT labelling reaction was quenched with the addition of 5% hydroxylamine and the samples were vortexed for 5 min and centrifuged at 8,000 rpm for 4 min at rt. An equal volume of supernatant from each condition was combined into a single Eppendorf. The combined peptides were fractionated (Pierce high pH Reversed-Phase

Peptide Fractionation kit) into 8 fractions and dried with a SpeedVac Vacuum concentrator. The dried fractions were resuspended in 2% acetonitrile and 0.5% trifluoroacetic acid in water to around 1 µg/µL.

LC-MS/MS and Data Analysis

Prepared peptide sample fractions were run using a Thermofisher Q-Exactive LC-MS/MS equipped with a Thermo EASY-SPRAY column as described previously.[2–4] The data obtained were processed with MaxQuant version 1.6.17.0, where peptides from the MS/MS spectra were searched in the human proteome database (UniProt accessed January 2021) and identified. The data were further analysed with Perseus 1.6.15.0, Microsoft Office Excel 365, and GraphPad Prism 9. A minimum of 2 unique peptides were selected for protein identification.
The mass spectrometry proteomics data have been deposited to the ProteomeXchange Consortium via the PRIDE[5] partner repository with the dataset identifier PXD036224.

Cell toxicity assay

MDA-MD-231 cells were seeded in a 96-well plate (7000 cells/well) and grown in low glucose DMEM with 10% FBS overnight. The next day, the media was replaced with media containing compound and SYTOX™ Green Nucleic Acid Stain (dye, 250 nM final concentration). For control, cells were treated with media containing 0.1% DMSO without dye, 0.1% DMSO with dye, 2 µg/mL puromycin and dye, and 2 µg/mL puromycin in 0.1% DMSO with dye. The cell plate was incubated at 37 °C with 5% CO_2 in the incubator. Phase and green fluorescence were imaged every 2 h for 3 days with the Incucyte Live-Cell Analysis Systems.

Supplementary Information: Photoswitching properties characterisation

General Methods

UV-Vis spectra were recorded on an Agilent Cary 60 UV-Vis spectrometer (wavelength range: 190 – 1100 nm, resolution: 1.5 nm, Light source: Xenon Flash Lamp 80 Hz) with a temperature controller. Polystyrene semi-micro cuvettes (1.5 mL, 1 cm) sealed with parafilm were used in the measurement. The data was analysed using WinUV software. LC-MS spectra were recorded on a Waters high-performance liquid chromatography (HPLC) system, including a 2767 autosampler, 515 pump, 2998 photodiode array (PDA) detector and a 3100-electrospray ionization (ESI) mass spectrometer, using MassLynx 4.1 software. Compounds were separated on a 4.6 mm × 100 mm analytical Waters XBridge C18 column using the following gradient: 20% to 98% acetonitrile in water with 0.1% formic acid over 12 min then 98% acetonitrile for 3 min. Two custom-made black boxes (WaveyTech Ltd) installed with 25 mW LED light bulbs of 340 nm, 365 nm, 405 nm, 450 nm and 457 nm wavelength were used to irradiate compounds.

Photostationary state (PSS) for linker **4** and **AP-PROTAC-1** was calculated with the UV-Vis-based Fischer's method.[6] **AP-PROTAC-1** ligand **JQ1-COOH** showed no absorption over the 280-600 nm UV-Vis range. For **AP-PROTAC-2**, the promiscuous kinase ligand **CTx-0294885** displayed an absorption centered around 340 nm (Figure S8). The **CTx-0294885** ligand and the photoswitchable linker absorb 365 nm light, which affects the calculation of PSS using UV-Vis. Therefore, LC-MS chromatograms of **AP-PROTAC-2** following irradiation were used to calculate the PSS.

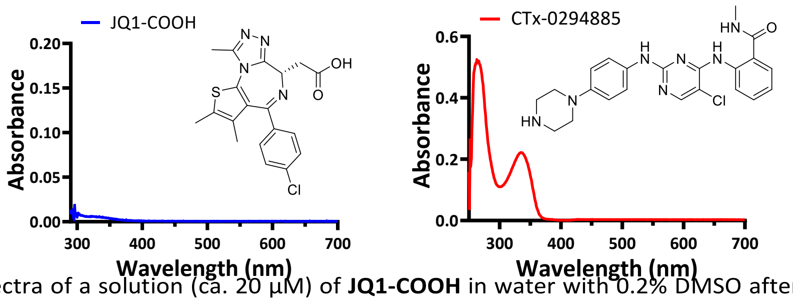

Figure S8. (A) UV-Vis spectra of a solution (ca. 20 µM) of **JQ1-COOH** in water with 0.2% DMSO after filtration with a 0.2 µm polyethersulfone (PES) filter. (B) UV-Vis spectra of a solution (ca. 10 µM) of **CTx-0294885** in DMSO.

UV-Visible spectroscopy determination of PSS

A solution of 20 µM linker **4** or **AP-PROTAC-1** in 0.2% DMSO in water was irradiated with 340 nm, 365 nm, 405 nm, 457nm LED for 90 s. A UV-Vis spectrum of the non-irradiated solution was recorded, and spectra following each irradiation were recorded. Using Fischer's method, the PSSs at 365 nm and 457 nm were calculated.

$$a_2 = (\frac{\Delta_1}{D_{\mathrm{E1}}} - \frac{\Delta_2}{D_{\mathrm{E2}}})/(1 + \frac{\Delta_1}{D_{\mathrm{E1}}} - n(1 + \frac{\Delta_2}{D_{\mathrm{E2}}})$$

$$a_1 = na_2, \mathrm{n} = \frac{D_{obsd\ 1_{\lambda max}} - D_{E_{\lambda max}}}{D_{\mathrm{obsd}\ 2_{\lambda max}} - D_{E_{\lambda max}}}$$

$$\Delta = D_{obsd} - D_E$$

Where a_1 and a_2 are the PSS ratios of the less stable *cis* (*Z*) isomer at wavelength 1 and 2. D_{obsd} is the observed absorbance, D_E is the absorbance of a solution containing only the more stable *trans* (*E*) isomer. Here, the spectrum of linker **4** or **AP-PROTAC-1** solution prepared freshly from a DMSO stock stored protected from light was assumed to contain only *trans* (*E*) isomer and used in the calculation. λ_{max} is the wavelength where the maximal absorption difference is found for *trans* (*E*) isomer and *cis* (*Z*) isomer.
Calculation details:
For linker **4**, λ_{max} was 341 nm.
To calculate PSS of linker **4** at 365 nm irradiation, for wavelength 1 (340 nm) and wavelength 2 (365 nm),

$$n = \frac{D_{obsd\ 1_{\lambda max}} - D_{E_{\lambda max}}}{D_{\mathrm{obsd}\ 2_{\lambda max}} - D_{E_{\lambda max}}} = \frac{0.00994 - 0.10608}{0.00983 - 0.10608} = 0.9989$$

$$\frac{\Delta_1}{D_{\mathrm{E1}}} = \frac{D_{obsd\ 1} - D_{E1}}{D_{\mathrm{E1}}} = \frac{0.01083 - 0.10685}{0.10685} = -0.8986$$

$$\frac{\Delta_2}{D_{\mathrm{E2}}} = \frac{D_{obsd\ 2} - D_{E2}}{D_{\mathrm{E2}}} = \frac{0.00323 - 0.06033}{0.06033} = -0.9465$$

$$a_2 = 0.999 \quad a_1 = na_2 = 0.998$$

Therefore, at 365 nm PSS there is 99% *Z*-**4** in the solution.

To calculate PSS of linker **4** at 457 nm irradiation, for wavelength 1 (450 nm) and wavelength 2 (457 nm),

$$n = \frac{D_{obsd\ 1_{\lambda max}} - D_{E_{\lambda max}}}{D_{\mathrm{obsd}\ 2_{\lambda max}} - D_{E_{\lambda max}}} = \frac{0.10683 - 0.11293}{0.10061 - 0.11293} = 0.495$$

$$\frac{\Delta_1}{D_{\mathrm{E1}}} = \frac{D_{obsd\ 1} - D_{E1}}{D_{\mathrm{E1}}} = \frac{0.01087 - 0.01053}{0.01053} = 0.0323$$

$$\frac{\Delta_2}{D_{\mathrm{E2}}} = \frac{D_{obsd\ 2} - D_{E2}}{D_{\mathrm{E2}}} = \frac{0.00788 - 0.00893}{0.00893} = -0.1177$$

$$a_2 = 0.251 \quad a_1 = na_2 = 0.124$$

Therefore, at 457 nm PSS there are 25% *Z*-**4** and 75% *E*-**4** in the solution.

For **AP-PROTAC-1**, λ_{max} was 344 nm.
To calculate PSS of **AP-PROTAC-1** at 365 nm irradiation, for wavelength 1 (340 nm) and wavelength 2 (365 nm),

$$n = \frac{D_{obsd\ 1_{\lambda max}} - D_{E_{\lambda max}}}{D_{\mathrm{obsd}\ 2_{\lambda max}} - D_{E_{\lambda max}}} = \frac{0.09770 - 0.20426}{0.08813 - 0.20426} = 0.9176$$

$$\frac{\Delta_1}{D_{\mathrm{E1}}} = \frac{D_{obsd\ 1} - D_{E1}}{D_{\mathrm{E1}}} = \frac{0.10268 - 0.20728}{0.20728} = -0.5046$$

$$\frac{\Delta_2}{D_{\mathrm{E2}}} = \frac{D_{obsd\ 2} - D_{E2}}{D_{\mathrm{E2}}} = \frac{0.04502 - 0.13437}{0.13437} = -0.6650$$

$$a_2 = 0.853,\ a_1 = na_2 = 0.783$$

Therefore, at 365 nm PSS there are 85% *Z* isomer, 15% *E* isomer in the solution.

To calculate PSS of **AP-PROTAC-1** at 457 nm irradiation, for wavelength 1 (405 nm) and wavelength 2 (457 nm),

$$n = \frac{D_{obsd\ 1_{\lambda max}} - D_{E_{\lambda max}}}{D_{\mathrm{obsd}\ 2_{\lambda max}} - D_{E_{\lambda max}}} = \frac{0.1556 - 0.20426}{0.17672 - 0.20426} = 1.767$$

$$\frac{\Delta_1}{D_{\mathrm{E1}}} = \frac{D_{obsd\ 1} - D_{E1}}{D_{\mathrm{E1}}} = \frac{0.02707 - 0.03337}{0.03337} = -0.1888$$

$$\frac{\Delta_2}{D_{\mathrm{E2}}} = \frac{D_{obsd\ 2} - D_{E2}}{D_{\mathrm{E2}}} = \frac{0.0191 - 0.01854}{0.01854} = 0.0302$$

$$a_2 = 0.217,\ a_1 = na_2 = 0.383$$

Therefore, at 457 nm PSS there are 22% *Z* isomer, 78% *E* isomer in the solution.

LC-MS spectra

UV traces of AP-PROTAC-1

Chromatograms of a 0.1 mM solution of AP-PROTAC-1 in water with 50% acetonitrile, non-irradiated and exposed to ambient light.

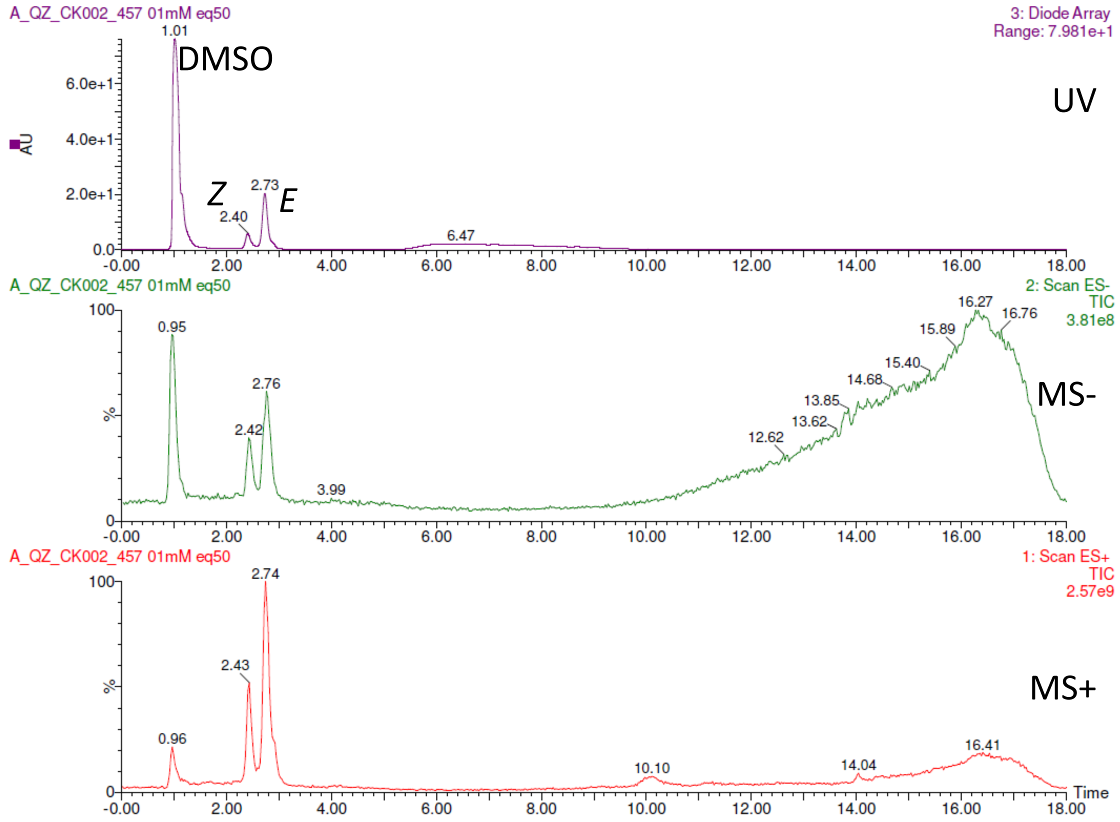

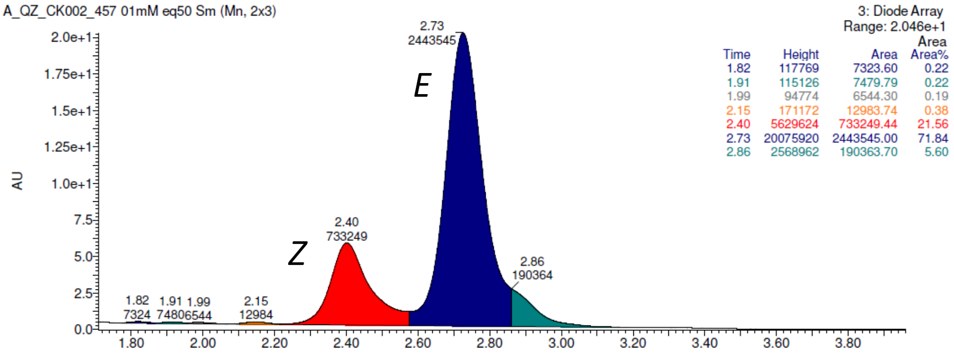

LC-MS method for PSS determination of AP-PROTAC-2

LC-MS chromatograms of a 0.1 mM solution of AP-PROTAC-2 in water with 20% acetonitrile, non-irradiated and exposed to ambient light.

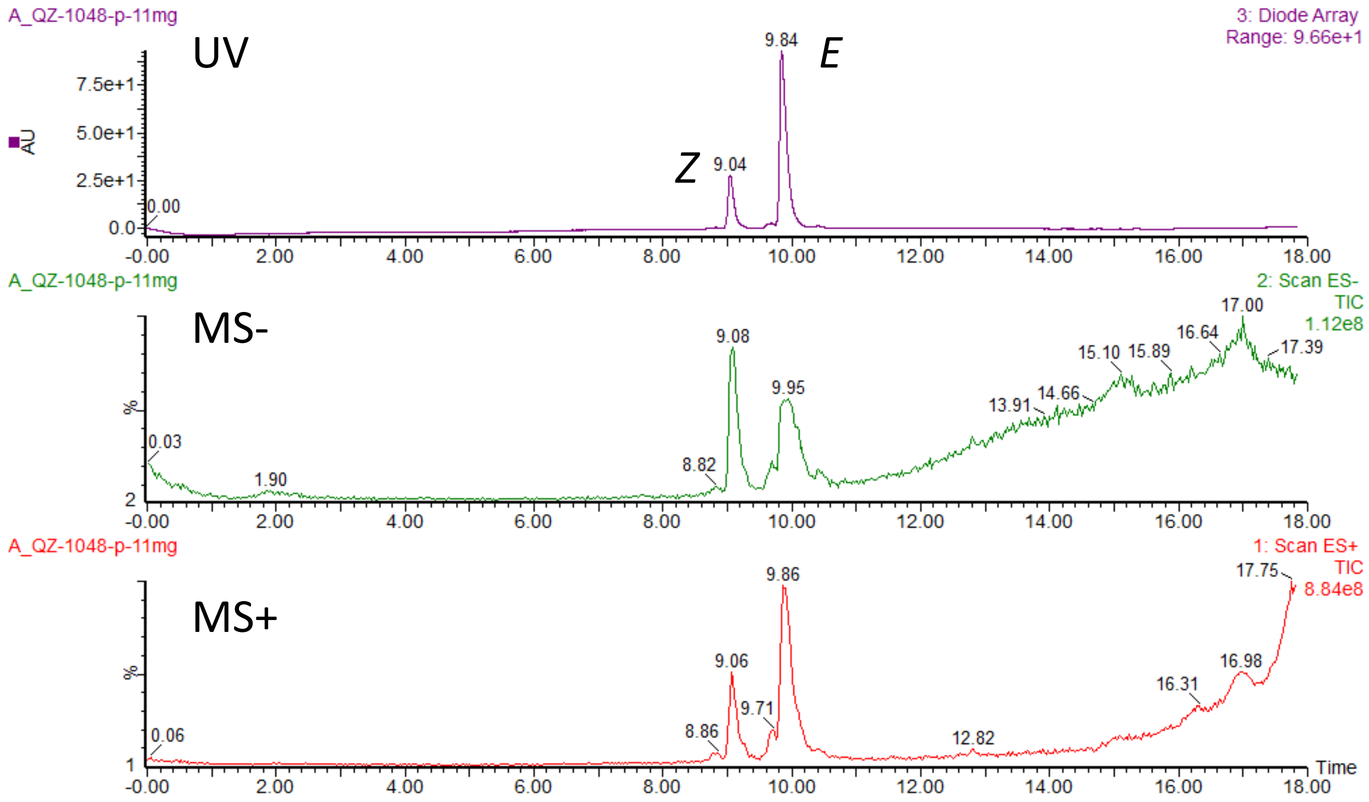

MS+ at 9.06 min and 9.91 min.

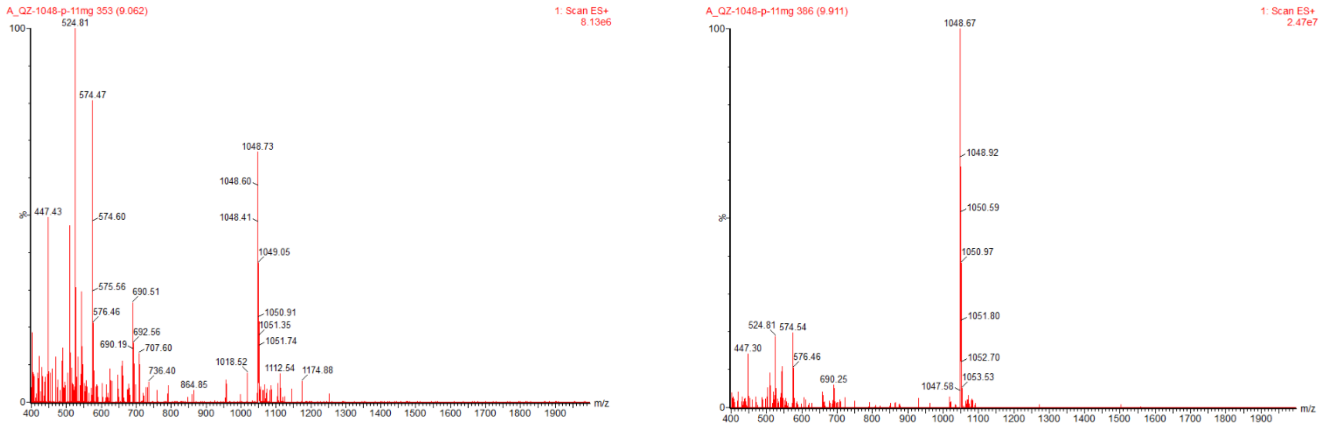

LC-MS chromatograms of AP-PROTAC-2, after 8 min of 457 nm irradiation.

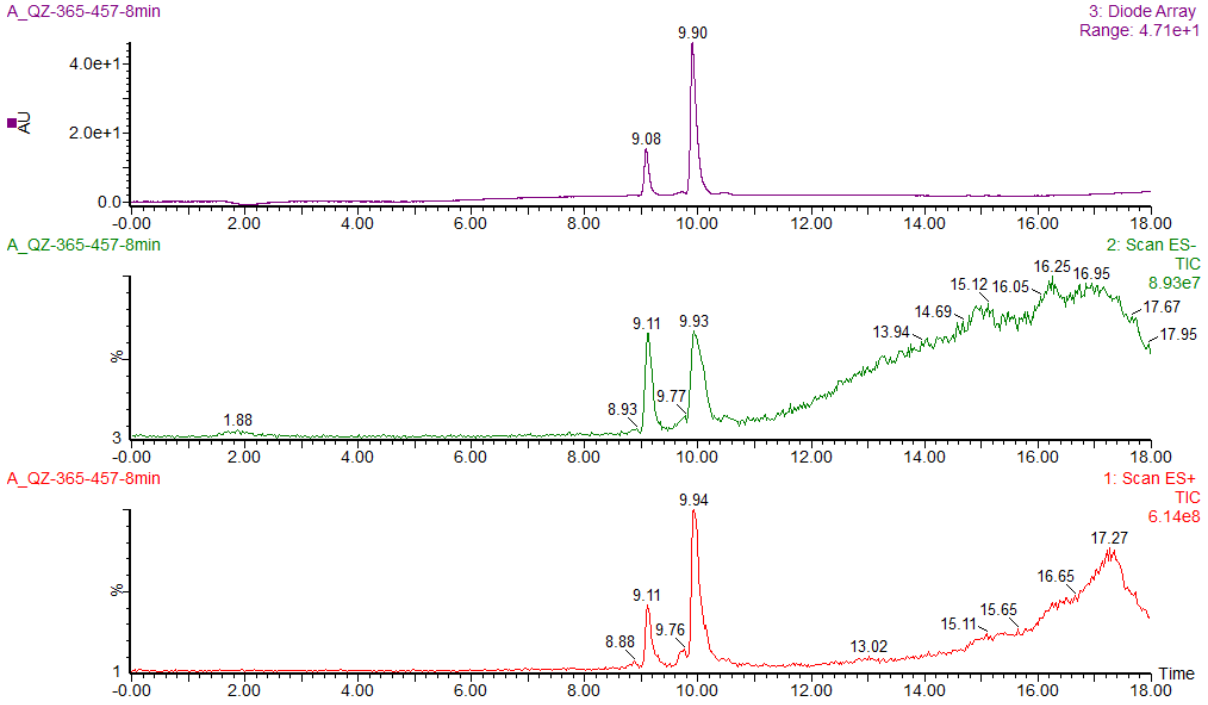

Area under curve of diode array showed PSS of 80% *E* and 20% *Z*.

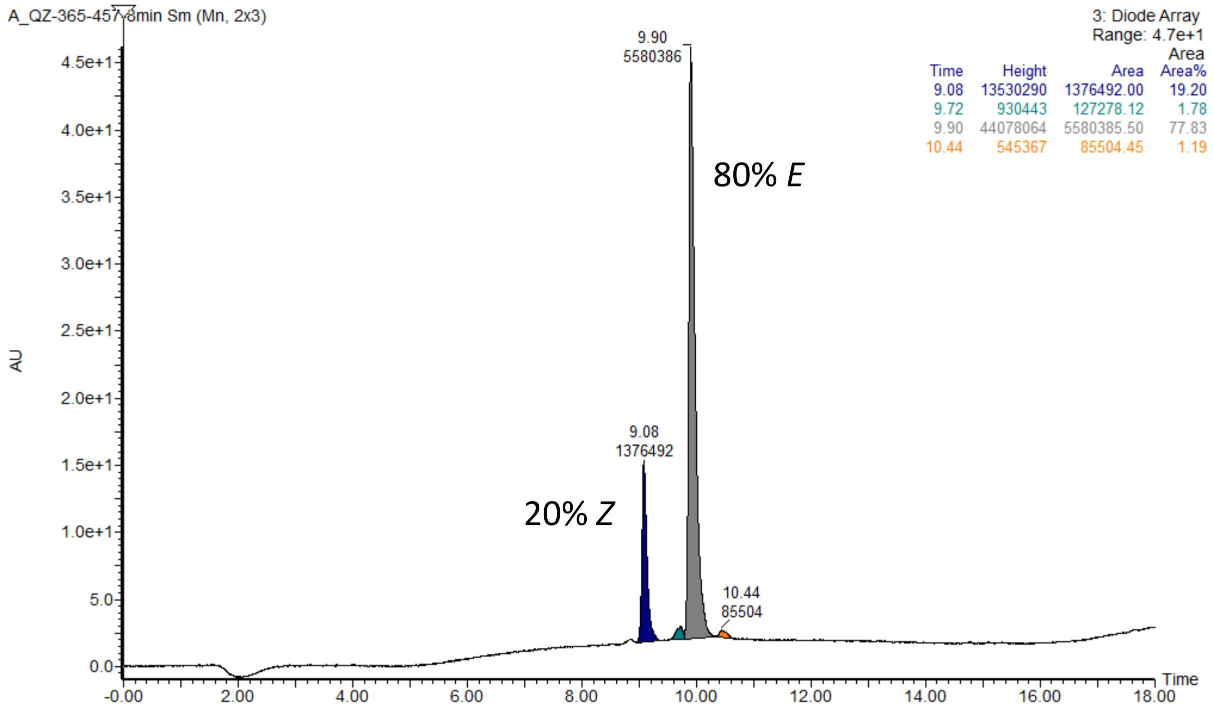

LC-MS chromatograms of AP-PROTAC-2, after 8 min of 457 nm irradiation and followed by 8 min of 365 nm irradiation.

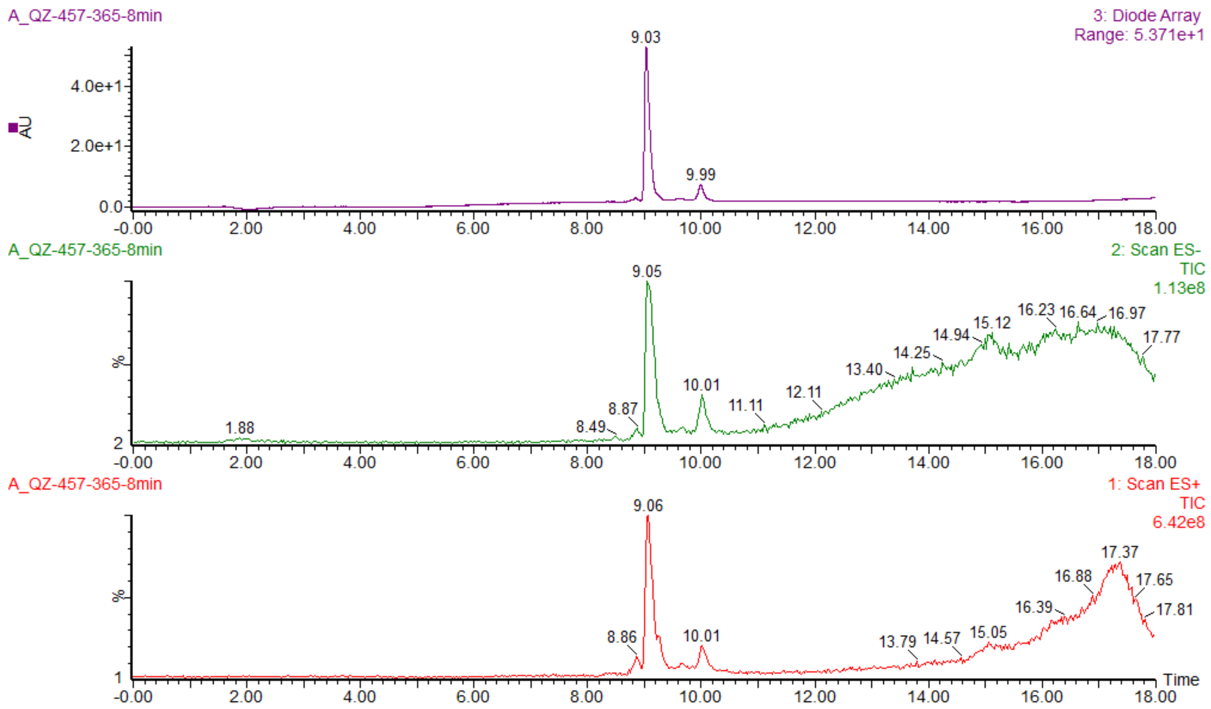

Area under curve of diode array showed PSS of 90% *Z* and 10% *E*.

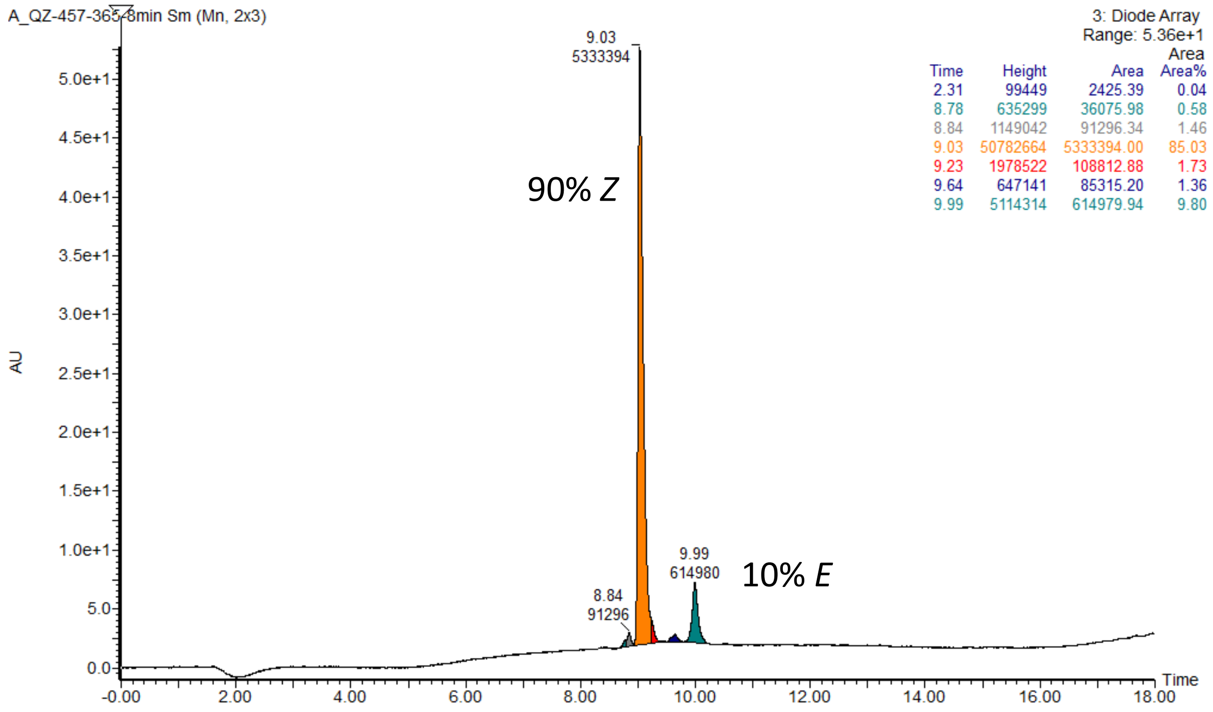

UV-Visible spectroscopy determination of thermal half-life

A solution of 12 µM **AP-PROTAC-2** in 0.1% DMSO in water was irradiated with 365 nm LED for 3 min. Following irradiation, the solution was kept in the UV-Vis spectrophotometer in a 37°C chamber for 24 h. A UV-Vis spectrum was recorded every 30 min during the incubation. It was assumed that the compound achieved the same conversion ratio with irradiation as observed in the LC-MS experiment (80% *E* with 457 nm and 90% *Z* with 365 nm irradiation).

The percentage of *Z* isomer was plotted against time (Figure S2), where an exponential trendline was fitted to the plot:

$[Z] = [Z]_0 e^{-kt},\ y = 0.847e^{-6.08\times10^{-6}x} \quad (\mathrm{R}^2 = 0.9707)$

From the rate constant k, the thermal half-life of the Z isomer could be calculated: $\mathrm{t}_{1/2} = \frac{ln2}{k} = 31.7\ h$

Supplementary Information: Computational modelling of PROTAC ternary complex

Our structural modelling approach partitions the PROTAC-complex sampling problem into several steps and starts with a template-based protein-small-molecule docking protocol ClusPro LigTBM, which orients PROTAC end-ligands with corresponding proteins.[7,8] Each PROTAC is bisected in the middle atom of the linker and conformers of the PROTAC halves have been generated by the ETKDG method and relaxed with MMFF (Figure S9).[9,10] The resulting half-PROTACs are aligned to the respective protein-ligand complex models and filtered for clashes. The middle atom positions of the remaining conformations have been projected to grids. Next, to find energetically favourable protein-protein complex poses, we use the FFT-based docking program PIPER with an additional "silent" term.[11–13] This term convolves the aforementioned grids and ensures that the middle atom on both sides of docking overlaps, thus it partially accounts for PROTAC accessible conformations and helps efficiently filter out unfeasible protein-protein complex poses. The structures where the half-PROTACs can be successfully connected into a complete PROTAC are relaxed by Amber energy minimization and clustered to produce complete ternary PROTAC complex models.[14] A pdb file of the representative ternary complex pose shown in the main text was provided as a separate file.

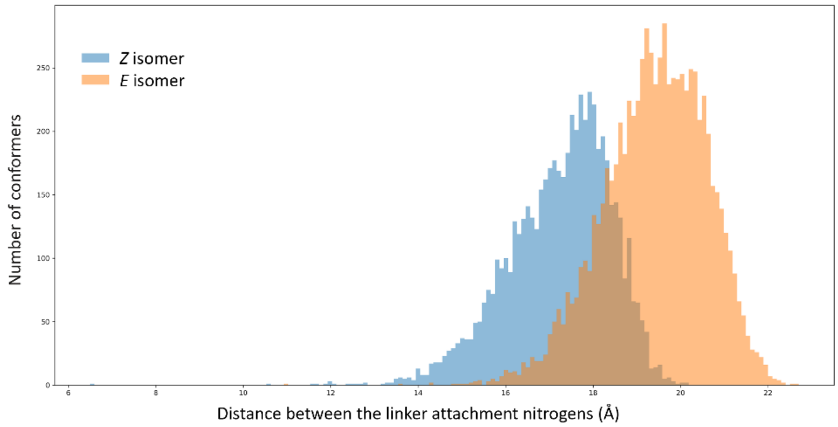

Figure S9. Histogram of the distances between the linker attachment nitrogens for *E* and *Z* ETKDG-derived MMFF-optimized PROTAC conformers. The distance between the attachment atoms among the top CRBN/(*E*)-AP-PROTAC-2/FAK complex models varies from 19.3 to 21.1 Å, which is barely reachable for *Z* isomer.

References

1 C. Russell, A. J. S. Lin, P. Hains, M. I. Simone, P. J. Robinson and A. Mccluskey, *RSC Advances*, 2015, **5**, 93433–93437.

2 M. H. Wright, B. Clough, M. D. Rackham, K. Rangachari, J. A. Brannigan, M. Grainger, D. K. Moss, A. R. Bottrill, W. P. Heal, M. Broncel, R. A. Serwa, D. Brady, D. J. Mann, R. J. Leatherbarrow, R. Tewari, A. J. Wilkinson, A. A. Holder and E. W. Tate, *Nature Chemistry*, 2014, **6**, 112–121.

3 M. H. Wright, D. Paape, H. P. Price, D. F. Smith and E. W. Tate, *ACS Infectious Diseases*, 2016, **2**, 427–441.

4 A. Mousnier, A. S. Bell, D. P. Swieboda, J. Morales-Sanfrutos, I. Pérez-Dorado, J. A. Brannigan, J. Newman, M. Ritzefeld, J. A. Hutton, A. Guedán, A. S. Asfor, S. W. Robinson, I. Hopkins-Navratilova, A. J. Wilkinson, S. L. Johnston, R. J. Leatherbarrow, T. J. Tuthill, R. Solari and E. W. Tate, *Nature Chemistry*, 2018, **10**, 599–606.

5 Y. Perez-Riverol, J. Bai, C. Bandla, D. García-Seisdedos, S. Hewapathirana, S. Kamatchinathan, D. J. Kundu, A. Prakash, A. Frericks-Zipper, M. Eisenacher, M. Walzer, S. Wang, A. Brazma and J. A. Vizcaíno, *Nucleic Acids Research*, 2022, **50**, D543–D552.

6 Ernst Fischer, *J. Phys. Chem.*, 1967, **71**, 3704–3706.

7 S. Kotelnikov, A. Alekseenko, · Cong Liu, · Mikhail Ignatov, D. Padhorny, E. Brini, · Mark Lukin, · Evangelos Coutsias, K. A. Dill and D. Kozakov, *Journal of Computer-Aided Molecular Design*, 2020, **34**, 179–189.

8 A. Alekseenko, S. Kotelnikov, M. Ignatov, M. Egbert, Y. Kholodov, S. Vajda and D. Kozakov, *Journal of Molecular Biology*, 2020, **432**, 3404–3410.

9 S. Riniker and G. A. Landrum, *Journal of Chemical Information and Modeling*, 2015, **55**, 2562–2574.

10 P. Tosco, N. Stiefl and G. Landrum, *Journal of Cheminformatics*, 2014, **6**, 37.

11 D. Kozakov, D. R. Hall, B. Xia, K. A. Porter, D. Padhorny, C. Yueh, D. Beglov and S. Vajda, *Nature Protocols*, 2017, **12**, 255–278.

12 D. Kozakov, R. Brenke, S. R. Comeau and S. Vajda, *Proteins: Structure, Function and Genetics*, 2006, **65**, 392–406.

13 D. Padhorny, A. Kazennov, B. S. Zerbe, K. A. Porter, B. Xia, S. E. Mottarella, Y. Kholodov, D. W. Ritchie, S. Vajda and D. Kozakov, *Proc Natl Acad Sci U S A*, 2016, **113**, E4286–E4293.

14 D. A. Case, T. E. Cheatham, T. Darden, H. Gohlke, R. Luo, K. M. Merz, A. Onufriev, C. Simmerling, B. Wang and R. J. Woods, *Journal of Computational Chemistry*, 2005, **26**, 1668–1688.

Author contributions

Conceptualisation, supervision and funding acquisition: EWT, JDH, MJF.
Funding acquisition: DK, AM.
Investigation, methodology, data curation, formal analysis: QZ, CSK, JRB, SK, MI.
Methodology, data curation, formal analysis, supervision: MM, DC, LZ, JLG, DK.
Conceptualisation, methodology: CPT.
Resources: JDH, JRB, AM, DK, JLG.
Writing – original draft, review and editing: QZ, CSK, SK.
Writing – review and editing: EWT, MJF, EDV, DK, MM, JLG.
All authors read and approved the final manuscript.

Acknowledgement

The authors thank Dr Charles Nicholas Saunders from WaveyTech Ltd for providing LED devices, GlaxoSmithKline Medicines Research Centre, Stevenage for providing reagents. The authors would like to thank Dr María Maneiro and Dr Ravi Singh for assistance with chemical synthesis, Miss Chotima Seripracharat for assistance with cell culture, Dr Wouter W. Kallemeijn, Dr Andrea Goya Grocin and Dr Jack Houghton for their assistance with the proteomics experiments. The authors would also like to thank Dr Lisa Haigh for assistance in acquiring high-resolution mass spectrometry (HRMS) and LC-MS/MS Orbitrap data, and Mr Peter R. Haycock for managing the Imperial College Chemistry Department NMR facility.

The authors acknowledge support from the Engineering and Physical Sciences Research Council (EPSRC) Grant (EP/R512540/1, EP/R00188X/1), GlaxoSmithKline Research and Development Ltd., The European Commission H2020 Marie Sklodowska Curie Actions Individual Fellowship grant, the Leverhulme Trust (RPG-2018-051), National Health and Medical Research Council (Australia), Cancer Research UK (Grant C24523/A25192), National Institutes of Health grants R01 GM140098 and RM1135136R01, and National Science Foundation grants DMS 2054251.

Original blots

AP-PROTAC-2 membrane cutting

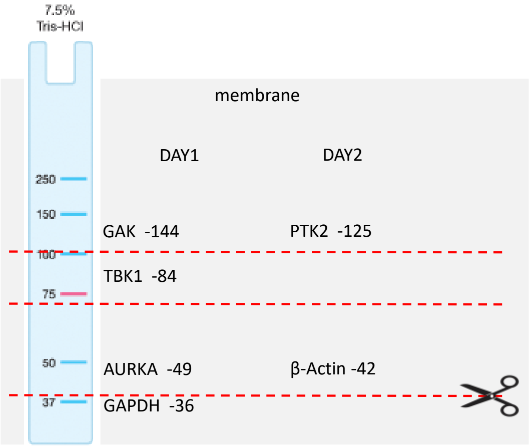

Figure 2D

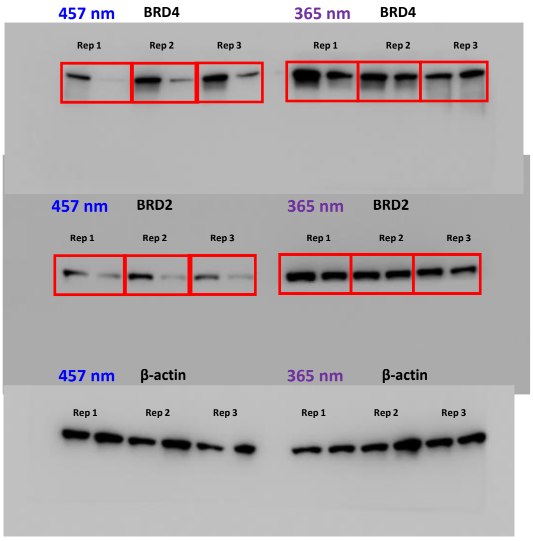

Figure 5

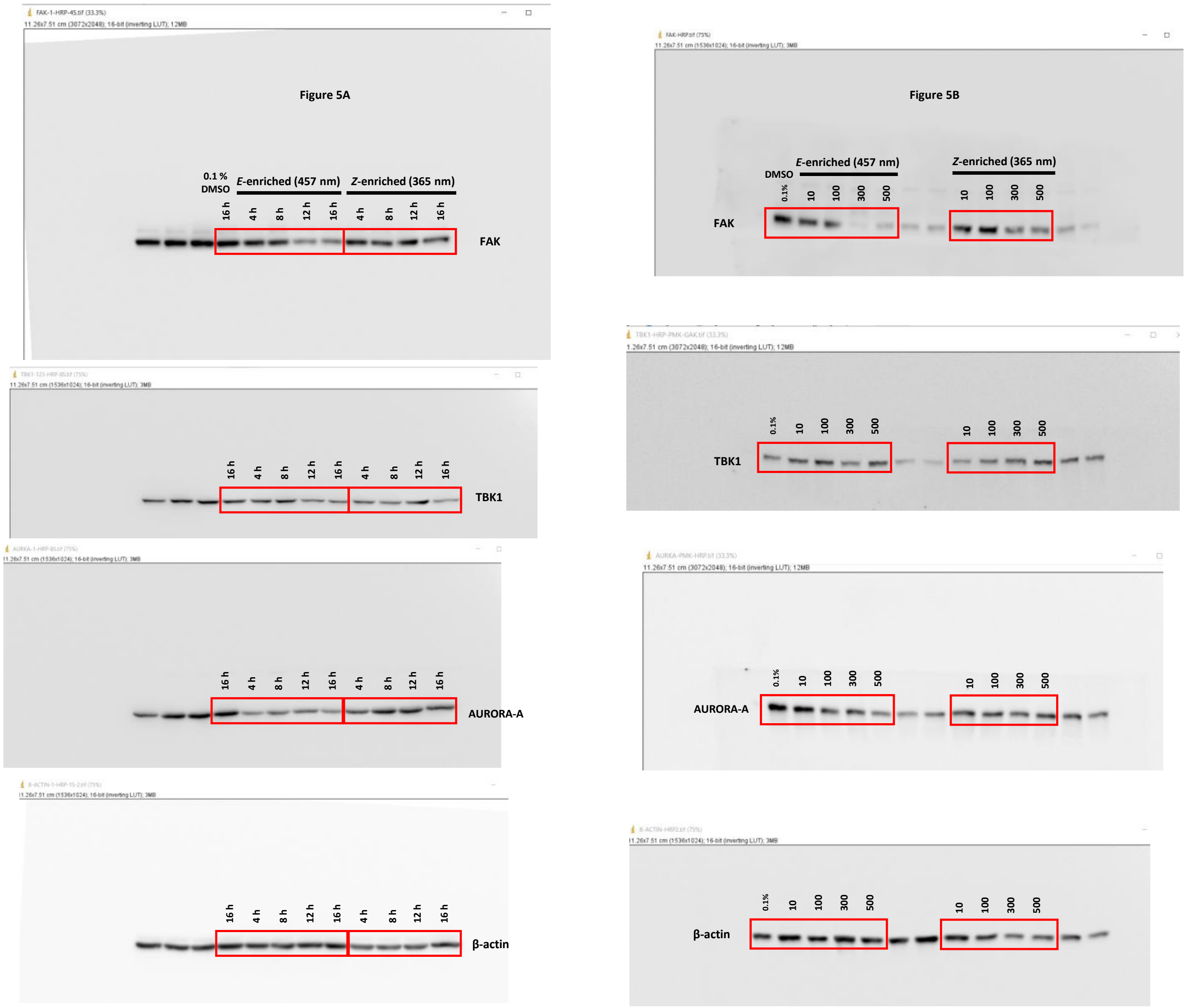

Note: Aurora-A was detected first, then the membrane was used for β-actin antibody incubation and HRP signal detection. In the blots of β-actin, weak bands above the main bands were signals from unstripped Aurora-A antibodies.

Figure S1

Figure S1A

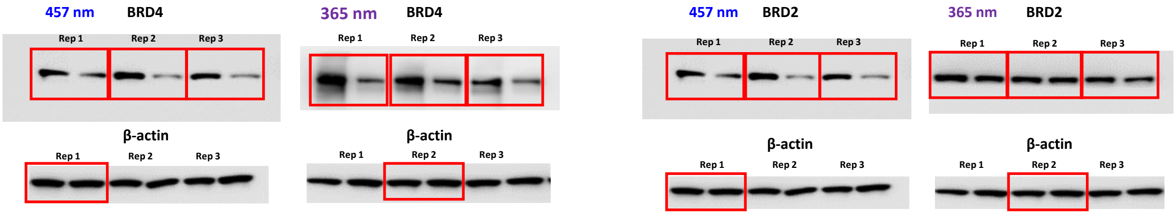

Figure S1B

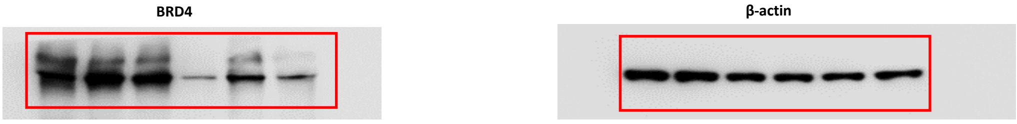

Figure S5

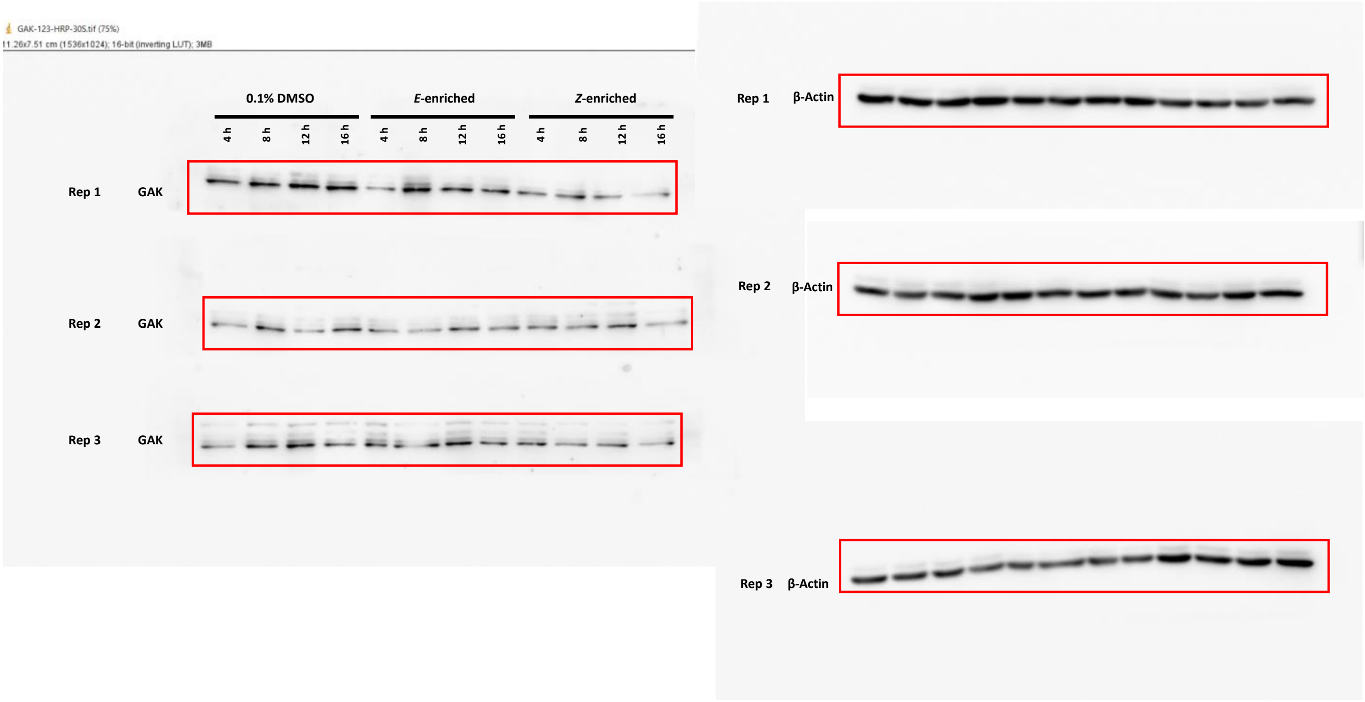

Figure S6C

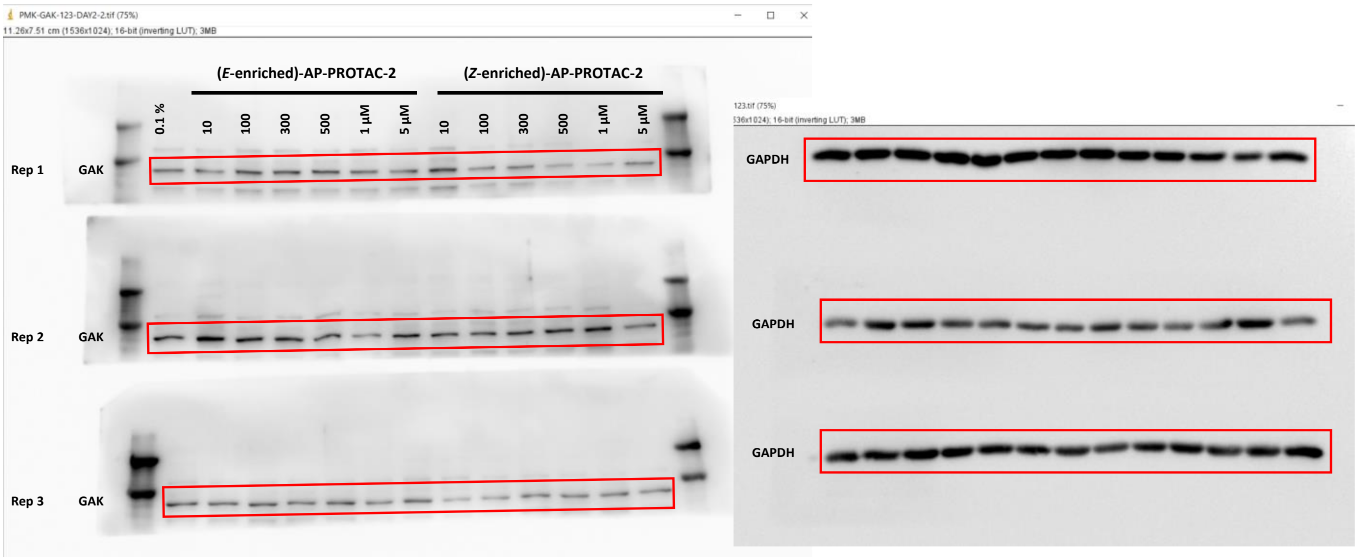

Figure S7

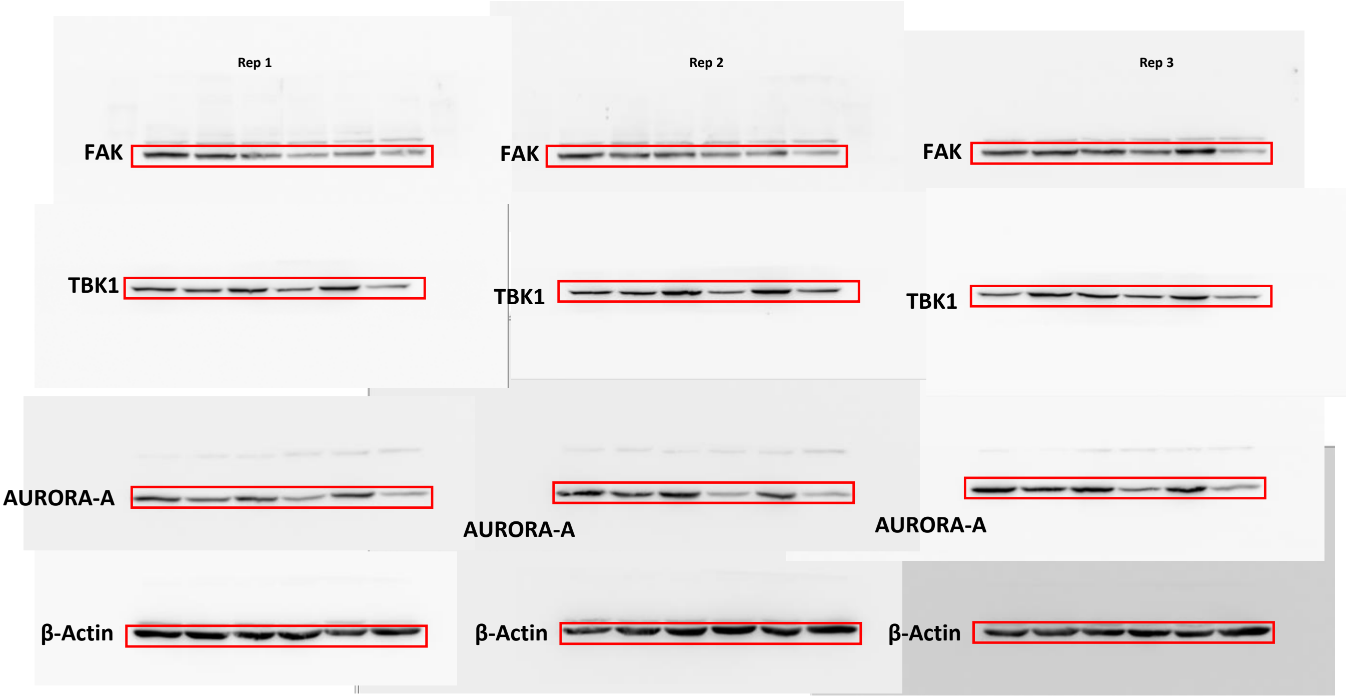

Supplement: CC-058-D2CC03092F-s002 [file CC-058-D2CC03092F-s002.pdf]
